# Alterations of gut microbiome accelerate multiple myeloma progression by increasing the relative abundances of nitrogen-recycling bacteria

**DOI:** 10.1186/s40168-020-00854-5

**Published:** 2020-05-28

**Authors:** Xingxing Jian, Yinghong Zhu, Jian Ouyang, Yihui Wang, Qian Lei, Jiliang Xia, Yongjun Guan, Jingyu Zhang, Jiaojiao Guo, Yanjuan He, Jinuo Wang, Jian Li, Jingchao Lin, Mingming Su, Guancheng Li, Minghua Wu, Lugui Qiu, Juanjuan Xiang, Lu Xie, Wei Jia, Wen Zhou

**Affiliations:** 1 State Key Laboratory of Experimental Hematology, Department of Hematology, Xiangya Hospital, Central South University, Changsha, Hunan China; 2 Key Laboratory for Carcinogenesis and Invasion, Chinese Ministry of Education, Key Laboratory of Carcinogenesis, Chinese Ministry of Health, China-Africa Research Center of Infectious Deseases, Cancer Research Institute, School of Basic Medical Sciences, Central South University, Changsha, Hunan China; 3 Shanghai Center for Bioinformation Technology, Shanghai Academy of Science and Technology, Shanghai, China; 4 Department of Hematology, Peking Union Medical College Hospital, Chinese Academy of Medical Sciences and Peking Union Medical College, Beijing, China; 5 Metabo-Profile Biotechnology (Shanghai) Co. Ltd., Shanghai, China; 6 State Key Laboratory of Experimental Hematology, Institute of Hematology & Blood Diseases Hospital, Chinese Academy of Medical Science & Peking Union Medical College, Tianjin, China; 7 School of Chinese Medicine, Hong Kong Baptist University, Kowloon Tong, Hong Kong, China

**Keywords:** Multiple myeloma, Gut microbiome, Nitrogen-recycling bacteria, Fecal microbiota transplantation

## Abstract

**Background:**

Gut microbiome alterations are closely related to human health and linked to a variety of diseases. Although great efforts have been made to understand the risk factors for multiple myeloma (MM), little is known about the role of the gut microbiome and alterations of its metabolic functions in the development of MM.

**Results:**

Here, in a cohort of newly diagnosed patients with MM and healthy controls (HCs), significant differences in metagenomic composition were discovered, for the first time, with higher bacterial diversity in MM. Specifically, nitrogen-recycling bacteria such as *Klebsiella* and *Streptococcus* were significantly enriched in MM. Also, the bacteria enriched in MM were significantly correlated with the host metabolome, suggesting strong metabolic interactions between microbes and the host. In addition, the MM-enriched bacteria likely result from the regulation of urea nitrogen accumulated during MM progression. Furthermore, by performing fecal microbiota transplantation (FMT) into 5TGM1 mice, we proposed a mechanistic explanation for the interaction between MM-enriched bacteria and MM progression via recycling urea nitrogen. Further experiments validated that *Klebsiella pneumoniae* promoted MM progression via de novo synthesis of glutamine in mice and that the mice fed with glutamine-deficient diet exhibited slower MM progression.

**Conclusions:**

Overall, our findings unveil a novel function of the altered gut microbiome in accelerating the malignant progression of MM and open new avenues for novel treatment strategies via manipulation of the intestinal microbiota of MM patients.

Video abstract.

## Introduction

Multiple myeloma (MM), the second most common hematological malignancy, has shown a uniformly increased incidence since 1990, especially in countries with middle and low-middle sociodemographic indices [1]. MM is characterized by the proliferation of malignant plasma cells in the bone marrow (BM) and the secretion of massive ineffective monoclonal immunoglobulin. The common manifestations of MM patients include hypercalcemia, renal damage, anemia, and bone lesions [2, 3]. Over the past decade, the introduction of novel agents such as proteasome inhibitors and immunomodulatory drugs has prolonged the survival of most MM patients, while MM remains incurable in the vast majority of cases [4, 5]. Infections are a major cause of morbidity and mortality in MM patients, and the risk of infection increases with active disease but decreases with responses to therapy [6].

Recently, emerging evidence revealed the underlying associations between gut microbiome and various diseases such as MM, colorectal cancer, and liver cirrhosis [7–9]. Notably, microbe-based strategies have been increasingly utilized, including the use of bacterial markers for clinical diagnosis and fecal microbiota transplantation (FMT) for disease treatment [8, 10, 11]. Empiric third-party FMT after allogeneic hematopoietic cell transplantation, which is the first-line strategy in MM treatment, appeared to be associated with an expansion of the recipient’s microbiome diversity [12]. Calcinotto et al. reported that *Prevotella heparinolytica* promoted the differentiation of Th17 cells colonizing the gut and migrating to the BM, where they favored the progression of MM in Vk*MYC mice, suggesting that commensal bacteria unleash a paracrine signaling network between innate and adaptive immunity to accelerate MM progression [7]. Additionally, compared with MM patients with minimal residual disease (MRD) positivity, the butyrate producer *Eubacterium hallii* possesses a higher relative abundance in MRD negativity, suggesting a potential link between microbiota composition and treatment responses in MM patients [13]. To date, however, the characterization of the gut microbiome and the interactions between the gut microbiome and metabolome in patients with MM have not been documented.

In this study, we aimed to fill this gap in knowledge and performed deep metagenomic sequencing of fecal samples from 37 participants, including newly diagnosed patients with MM and healthy controls (HCs). We discovered a significant difference in bacterial composition between the two groups, and enrichment of nitrogen-recycling bacteria in MM. Also, the functional alterations of the gut microbiome and the metabolic correlation between MM-enriched bacteria and host metabolomics profiling suggested that the altered gut microbiota in MM were predominantly involved in nitrogen recycling and utilization. In subsequent FMT experiments, we found that the mice with fecal microbiota from MM patient showed significantly accelerated progression of MM tumors, which was associated with the biosynthesis of l-glutamine from MM-enriched bacteria in the host. Further experiments showed that *Klebsiella pneumoniae* by gavage facilitated MM progression in vivo, while *glnA*-mutation *Klebsiella pneumoniae* had no effect. In addition, slower MM progression was discovered in mice fed with a glutamine-deficient diet. Our findings imply a broad mechanism involved in nutrient conservation and provide new insights for MM treatment in the future.

## Results

### Greater bacterial diversity in patients with MM

To explore the potential connection between the gut microbiome and MM, we performed shotgun metagenomic sequencing of fecal samples from 19 patients with newly diagnosed MM and 18 gender- and age-matched HCs. The sequences were curated and used for alignment and classification using Kraken and standard Kraken database [14]. In this study, only the bacterial community was taken into consideration. The majority of bacterial read counts in the cohort were dominated by the phyla *Bacteroidetes* and *Firmicutes*, representing 74% and 14% of the microbes, respectively, followed by *Proteobacteria* (9%) and *Actinobacteria* (2%). Thus, about 99% of bacterial read counts were covered by the four most abundant phyla in both HC and MM groups (Figure S1a, Additional file 1). Meanwhile, the *Proteobacteria* was more abundant in MM (Figure S1b, Additional file 1). Furthermore, about 96% of the microbes were accounted for by the thirty most abundant genera. Statistically, when compared with HC subjects, eight of those genera were enriched in MM, including *Citrobacter*, *Enterobacter*, *Klebsiella*, *Streptococcus*, *Collinsella*, *Intestinimonas*, *Veillonella*, and *Odoribacter* (Figure S2, Additional file 2). We further investigated the microbial co-occurrence network between the top 30 genera in the whole subjects (HC&MM), HC subjects and MM subjects, respectively (Figure S3a-c, Additional file 3; Table S1a-c, Additional file 4). Interestingly, we found more plentiful interactions between genera in HC than in MM, suggesting that rich interactions were perhaps necessary to maintain balance in the variety and complexity of the intestinal flora. In contrast, poor interactions in MM suggested that the robustness of gut microbiome was downregulated and that some bacteria with low node degree was susceptible to the change in the intestinal environment, such as *Citrobacter*, *Enterobacter*, and *Klebsiella* (Figure S3c, Additional file 3**)**.

**Fig. 1:**
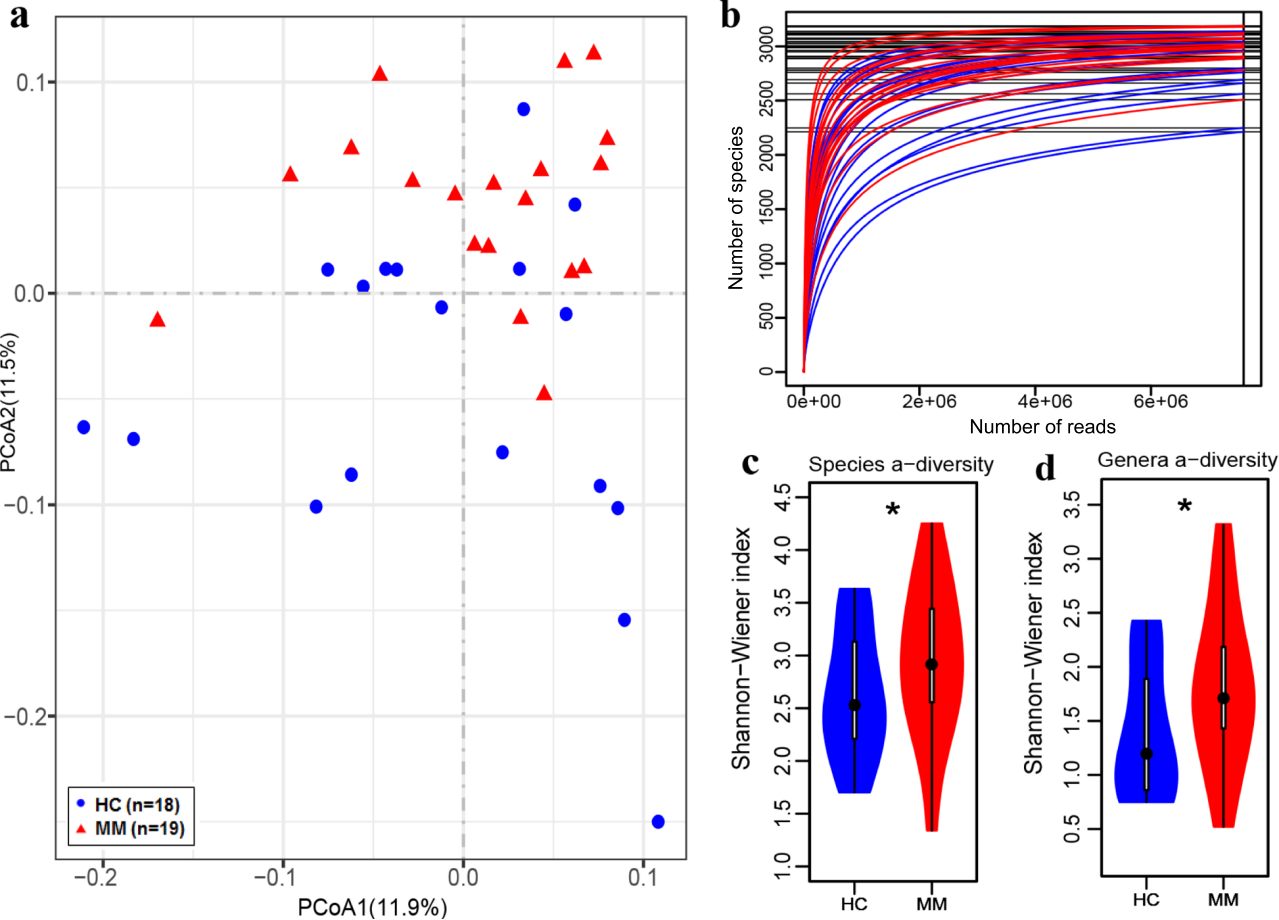
Gut microbiome characterization and bacterial diversity. **a** PCoA based on the Bray-Curtis dissimilarity index shows the between-subjects (β) diversity across groups, in which the blue circles and red triangles represent HC and MM subjects, respectively (PERMANOVA, *P =* 0.001). **b** Species rarefaction curves in blue or red indicate HC and MM subjects, respectively. **c**, **d** Violin graphs in blue or red show the Shannon-Wiener index of HC and MM subjects, respectively, at the species level (**c**) and at the genus level (**d**) (one-tailed Wilcoxon rank-sum test, *P =* 0.045 at the species level and *P =* 0.043 at the genus level)

We next analyzed the abundance of species in the cohort statistically. Primarily, principal coordinate analysis (PCoA) based on the Bray-Curtis dissimilarity index was performed to detect the significant difference in bacterial composition between MM samples and controls (Fig. 1a). The species rarefaction curve for each sample was performed and was found to approach saturation, indicating that the sequencing depth was adequate (Fig. 1b). In addition, we speculated that MM samples represented by red rarefaction curves exhibited higher species richness [15]. Indeed, the higher bacterial diversity in MM was confirmed by the greater Shannon-Wiener index at both the species (Fig. 1c) and genus (Fig. 1d) levels. Similarly, patients with colorectal cancer and liver cancer were reported increased microbial diversity [11, 16]. Thus, greater bacterial diversity is probably not an indication of healthy gut microbiota in this cohort.

### Nitrogen-recycling bacteria were significantly enriched in MM

**Fig. 2:**
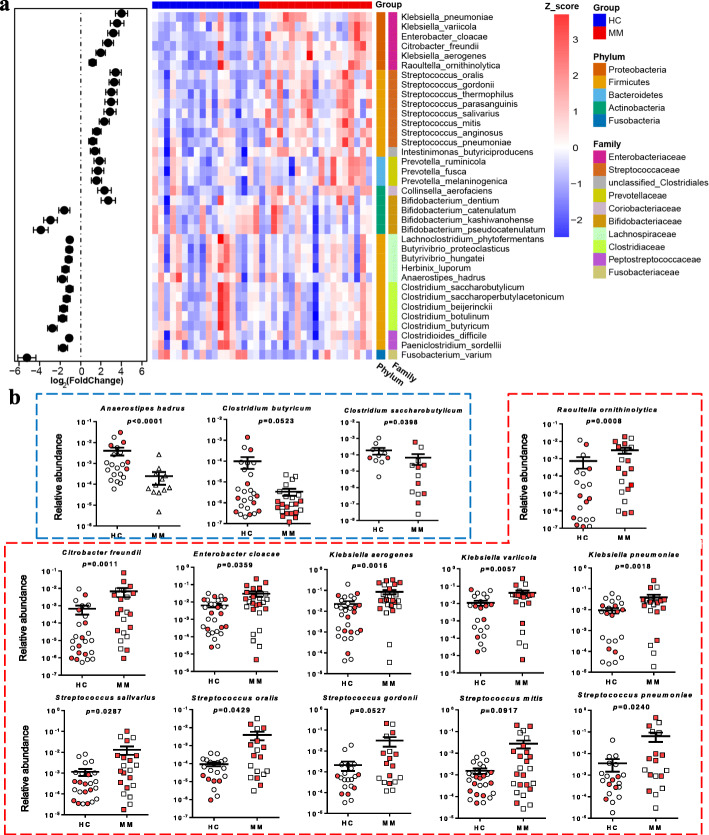
Species with differential abundance in HC and MM subjects of the cohort. **a** Left panel: the fold change of abundance of 36 different species in MM in relation to HC. Right panel: heatmap shows the scaled logarithm base-10 of the abundance of 20 MM-enriched species and 16 HC-enriched species. **b** Graphs indicate the species with significant differential abundance in an expanded cohort as verified using qPCR. HC-enriched species are highlighted in blue frame, while MM-enriched species in red frame. In HC, circles in red and white represent the subjects from a new collection of controls and metagenomic sequenced group, respectively. In MM, squares in red and white represent the subjects from a new collection of MM patients and metagenomic sequenced group, respectively. *P* value was determined by using two-tailed Mann-Whitney test. Note that some species were undetected in some samples in Fig. 2b. There are 6 and 4 undetected samples for *Anaerostipes hadrus* and *Clostridium saccharobutylicum*, respectively. And, there are 9, 3, 2, and 3 undetected samples for *Raoultella ornithinolytica*, *Citrobacter freundii*, *Klebsiella variicola*, and *Klebsiella pneumoniae*, respectively

To further identify the species with differential abundances, we performed differential analysis based on the aligned read counts of species using DESeq2 [17]. As a result, 60 differential species and subspecies were identified (Table S2, Additional file 5). In this study, we focused on those species with adjusted *P* value of less than 0.01, including 20 MM-enriched species and 16 HC-enriched species (Fig. 2a). Furthermore, their relative abundances were determined in an expanded cohort using qPCR of 16S rDNA, confirming that most of the tested species had a consistent trend with the aforementioned metagenomic analysis (Fig. 2b; Figure S4, Additional file 6). In particular, as shown in Fig. 2b, *Anaerostipes hadrus*, *Clostridium butyricum*, and *Clostridium saccharobutylicum*, belonging to *Clostridiales*, were more abundant in HC than in MM. Indeed, these three species play a significant role in gut health, contributing to the production of short-chain fatty acids [18–21]. In contrast, eleven opportunistic pathogens were enriched in MM, including *Raoultella ornithinolytica*, *Citrobacter freundii*, *Enterobacter cloacae*, *Klebsiella aerogenes*, *Klebsiella variicola*, *Klebsiella pneumoniae*, *Streptococcus salivarius*, *Streptococcus oralis*, *Streptococcus gordonii*, *Streptococcus mitis*, and *Streptococcus pneumoniae*. These bacteria belong to the *Enterobacteriaceae* or *Streptococcaceae* based on the taxonomic classification (Figure S5a, Additional file 7), and their abundances were higher in MM patients with ISS-III than in MM patients with ISS-II (Figure S5b, Additional file 7).

Interestingly, *Enterobacteriaceae* and *Streptococcaceae* (*Streptococcus*) are reportedly enriched in patients with ulcerative colitis [22]. Furthermore, a previous study demonstrated that nitrogen fixation occurs in fruit flies, resulting from the activity of stable and dominant populations of *Enterobacteriaceae* located in the gut, such as *Citrobacter freundii*, *Klebsiella* sp., and *Enterobacter* sp. [23]. Several uricolytic bacteria isolated from the gut of termites are assigned to *Streptococcus* sp. and *Enterobacteriaceae* [24–26]. The fact that gut bacteria recycle nitrogen from uric acid in termites is considered as a strategy for nutrient conservation [27]. In addition, many ureolytic bacteria are known to recycle nitrogen from urea in the gastrointestinal tract of ruminant and monogastric animals, such as *Streptococcus* sp. [28] In sum, several nitrogen-recycling bacteria are significantly enriched in MM subjects in this cohort.

### MM-enriched bacteria result from the accumulated urea nitrogen in MM

**Fig. 3:**
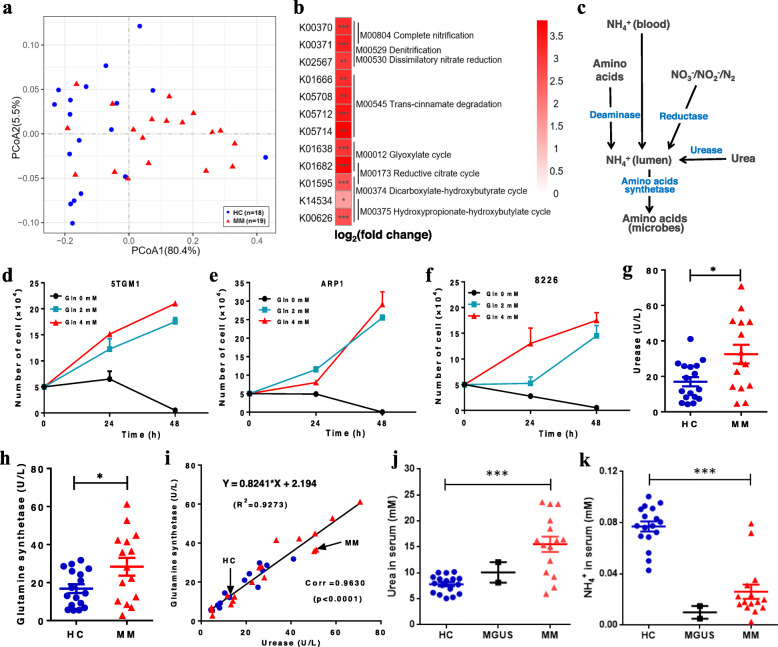
Functional alterations of microbes (KOs) in MM. **a** PCoA based on Bray-Curtis dissimilarity index shows the between-samples (β) diversity (PERMANOVA, *P* = 0.004). **b** Eight modules mapped by at least 2 identified KOs. **c** Metabolic pathways of NH_4_^+^ in the intestinal lumen. **d**–**f** Cell proliferation curves with different glutamine concentrations, including 5TGM1 (**d**), ARP1 (**e**), and 8226 (**f**). **g**, **h** The activities of urease (**g**) and glutamine synthase (**h**) in HC and MM subjects, respectively. **i** Linear fitting of urease and glutamine synthase activity in all subjects, in which blue dots and red triangles represent HC and MM samples, respectively. **j**, **k** The concentrations of serum urea (**j**) and NH_4_^+^ (**k**) in HC, MGUS, and MM samples, respectively. *P* value was determined by using two-tailed unpaired *t* test. *P < 0.05, **P < 0.01, ***P < 0.001

To dissect the biological functions of the gut microbiome, we aligned the clean sequences to the Kyoto Encyclopedia of Genes and Genomes (KEGG) using Metagenome Composition Vector (MetaCV) [29]. PCoA based on the Bray-Curtis dissimilarity index of KEGG orthology (KO) revealed the difference in functions between MM subjects and controls (Fig. 3a). Subsequently, based on the read counts across the two groups, 232 differential KOs were identified using DESeq2 (Table S3, Additional file 8). Interestingly, all of the identified KOs were enriched in MM. It appears logical to consider that the identified KOs reflect the functions of the more abundant microbes in MM. Based on the identified differential KOs, eight modules were mapped (Fig. 3b), including complete nitrification (M00804), denitrification (M00529), dissimilatory nitrate reduction (M00530), trans-cinnamate degradation (M00545), glyoxylate cycle (M00012), reductive citrate cycle (M00173), dicarboxylate-hydroxybutyrate cycle (M00374), and hydroxypropionate-hydroxybutyrate cycle (M00375). The modules M00804, M00529, and M00530 are involved in nitrification and denitrification of the nitrogen cycle. Additionally, a prior report showed the limitation of total nitrogen in the mammalian large intestine can enable hosts to regulate the microbial communities [30]. Here, we speculated that the dominant microbes in MM may result from the accumulation of host-secreted nitrogen.

Indeed, the gastrointestinal tract [31–34] is thought to be the indispensable site for ammonia (NH_4_^+^) recycling and biosynthesis of amino acids including the essential amino acids. The NH_4_^+^ in the intestinal lumen may be derived from blood-NH_4_^+^ transport, amino acid deamination, urea hydrolysis, and reduction of other nitrogen-containing compounds (Fig. 3c). Previous studies have shown that both human myeloma cell lines (HMCLs) and primary BM CD138(+) cells expressed high-level glutamine transporters and negligible glutamine synthetase (GS) activity, relied heavily on extracellular glutamine uptake, and produced excess NH_4_^+^ in the presence of glutamine [35]. Accordingly, we cultured mouse 5TGM1 MM cells and human ARP1 and 8226 MM cells, all of which showed proliferation proportional to the concentration of l-glutamine (Fig. 3d–f). Furthermore, we detected moderate elevation of urease (URE) and GS activities in MM feces (Fig. 3 g, h) and found a positive correlation between URE and GS activities for each sample, including HC and MM subjects (Fig. 3i). The results implied that the MM-enriched bacteria contributed to the elevated urea hydrolysis and glutamine synthesis in MM. In addition, we detected substantially more urea and less NH_4_^+^ in the serum of MM than HC subjects, while the levels in two monoclonal gammopathy of undetermined significance (MGUS) samples had values that were in between (Fig. 3j, k) because MGUS is a precursor state of MM tumorigenesis. Thus, the urea that is initially accumulated in the serum of MM subjects may eventually enter the intestinal tract, causing the enrichment of the aforementioned microorganisms in the intestinal tract.

### MM-enriched bacteria were associated with distinct metabolic modes in MM

**Fig. 4:**
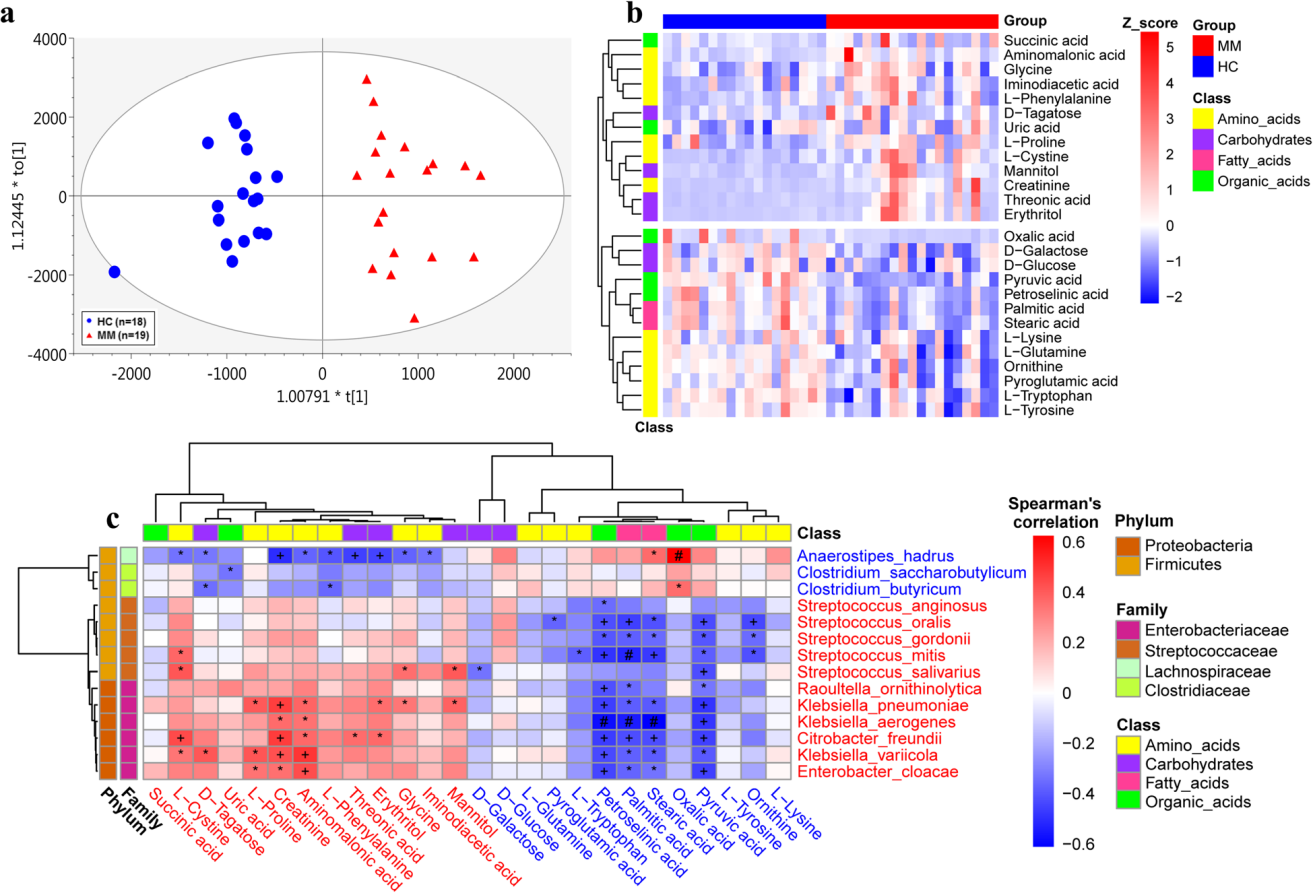
Metabolic profiling of host serum. **a** OPLS-DA score plot based on the metabolic profiling of serum samples, in which blue dots and red triangles represent HC and MM subjects, respectively (R2Y = 0.853; Q2 = 0.679). **b** Heatmap shows the scaled abundance of 26 differential metabolites. **c** Heatmap illustrates the Spearman’s correlation between 14 differential species and 26 differential metabolites, in which the red and blue species and metabolites denote enrichment in MM and HC samples, respectively. *, +, and # all suggest the significance, *P < 0.05, +*P* < 0.01, #*P* < 0.001

To further probe the alterations of microbe-host interactions in MM, we examined the metabolic profiling in the fasting serum of hosts. This is based on the notion that the metabolites and fermentation products in intestinal flora can enter the bloodstream and exert functional effects on the physiology of the host. Based on the abundance of metabolites detected with untargeted metabolomics, we performed orthogonal projections to latent structures discriminant analysis (OPLS-DA). The samples from distinct groups were largely separable according to the score scatter plot, suggesting distinct metabolic modes (Fig. 4a). Moreover, as depicted in Fig. 4b, there were 26 statistically differential metabolites, including 13 MM-enriched and 13 MM-depleted metabolites, most of which were classified as amino acids [36]. Notably, high-level creatinine has been used previously as an important clinical indicator because renal impairment is one of the most common symptoms of patients with MM [37, 38].

Since the metabolites listed in Fig. 4b may be fermented metabolites produced by the gut microbiota, we calculated the Spearman’s correlation between the 26 differential metabolites and the 14 validated differential species (Fig. 4c, the correlation with enlarged differential species is presented in Figure S6, Additional file 9). We found that the HC-enriched species correlated positively with HC-enriched metabolites but negatively with the MM-enriched metabolites. Consistently, the MM-enriched species correlated positively with MM-enriched metabolites but negatively with HC-enriched metabolites, implying highly uniform metabolic interactions between gut microbiota and the host. Here, no species were identified to correlate with l-glutamine with statistical significance, and this likely resulted from heavy consumption of l-glutamine by the host. In contrast, pyruvic acid, stearic acid, palmitic acid, and petroselinic acid correlated negatively with most MM-enriched species, while creatinine, aminomalonic acid, and l-proline were positively associated with a number of MM-enriched species, such as *Enterobacter cloacae*, *Klebsiella pneumoniae*, and *Klebsiella variicola* (Fig. 4c).

### MM-enriched bacteria facilitate MM progression by recycling urea nitrogen in mice

**Fig. 5:**
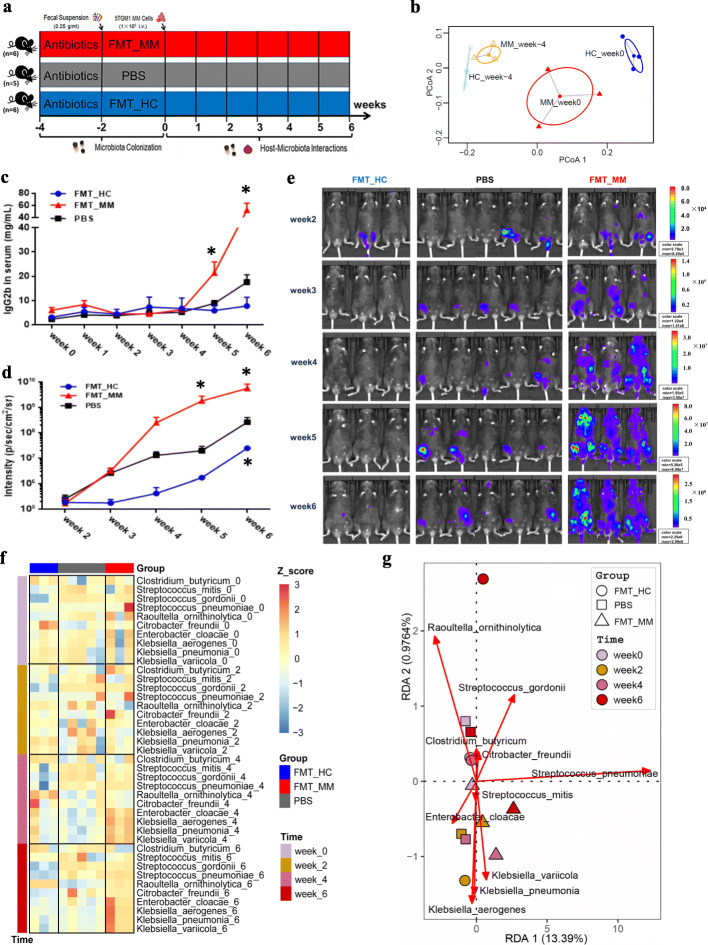
FMT experiment with MM and HC feces. **a** Schematic representation of FMT experiments. **b** Based on the Euclidean distance calculated from the relative abundances of several characteristic bacteria, experiments with PCoA show that these bacteria changed in FMT_HC and FMT_MM mice between week -4 and week 0. **c** Lines in blue, in red, and in black denote the concentration of serum lgG2b in FMT_HC, FMT_MM, and PBS mice, respectively. **d** Lines in blue, in red, and in black indicate the fluorescence intensity of live imaging in FMT_HC, FMT_MM, and PBS mice, respectively. **e** Live imaging of all recipient mice. **f** Heatmap reflects the changes of the scaled relative abundance of bacteria over time in FMT_HC, FMT_MM, and PBS mice. **g** Biplot of RDA of the bacteria in FMT_HC, FMT_MM, and PBS mice. *P* value was determined by two-way ANOVA test. *P < 0.05, **P < 0.01, ***P < 0.001

To validate the function of the microbe-host interactions in MM progression, we performed FMT experiments by using oral gavage in mice (Fig. 5a). 5TGM1 mice [39] aged 8~12 weeks were selected and treated with a cocktail of broad-spectrum antibiotics in the drinking water to establish flora-deficient mice and facilitate colonization of fecal microorganisms [40]. All mice were randomly divided into three groups, namely FMT_MM, FMT_HC, and PBS. As shown in Fig. 3i, we randomly selected one fecal donor for FMT_MM and FMT_HC groups, respectively. At week -4 and week 0, the relative abundances of several characteristic species were separately measured in the feces of FMT_MM and FMT_HC mice, respectively. Based on the Euclidean distance calculated from the relative abundances of these species, PCoA was performed to illustrate the distinct colonization of microflora in FMT_MM and FMT_HC mice (Fig. 5b).

Next, we induced mice to develop MM via tail-vein injection of 5TGM1 MM cells that were validated to require large amounts of exogenous l-glutamine (Fig. 3d). MM progression in the mice was monitored weekly by measuring tumor burden, including the concentration of IgG2b (Fig. 5c) and live imaging of animals (Fig. 5d, e). We found that over time, the tumor fluorescence intensity of all mice increased steadily. At week 6, the tumor-associated fluorescence intensity in FMT_MM mice was markedly higher than that of PBS mice, while the intensity in FMT_HC mice was lower than that of PBS mice. In addition, the concentration of immunoglobulin (IgG2b) sharply differed among the three groups. In the meantime, we also measured the relative abundances of several characteristic species every other week to monitor the alterations of the gut microbiome in mouse feces. We found that the relative abundances of most MM-enriched bacteria were the highest in FMT_MM mice, followed by PBS mice, and the lowest in FMT_HC mice, while the differences were increasingly striking from week 0 to week 6 (Fig. 5f; Figure S7, Additional file 10). In keeping with these findings, redundancy analysis (RDA) also revealed time-dependent variations among the three groups of mice (Fig. 5g). The distribution of the three groups was the most discrete at week 6: the FMT_MM group located at the bottom of the RDA2 while the FMT_HC group and the PBS group located at the top. Meanwhile, the FMT_MM mice appeared to be characterized by *Klebsiella pneumoniae*, *Klebsiella aerogenes*, and *Klebsiella variicola* while FMT_HC mice were characterized by *Clostridium butyricum*.

**Fig. 6:**
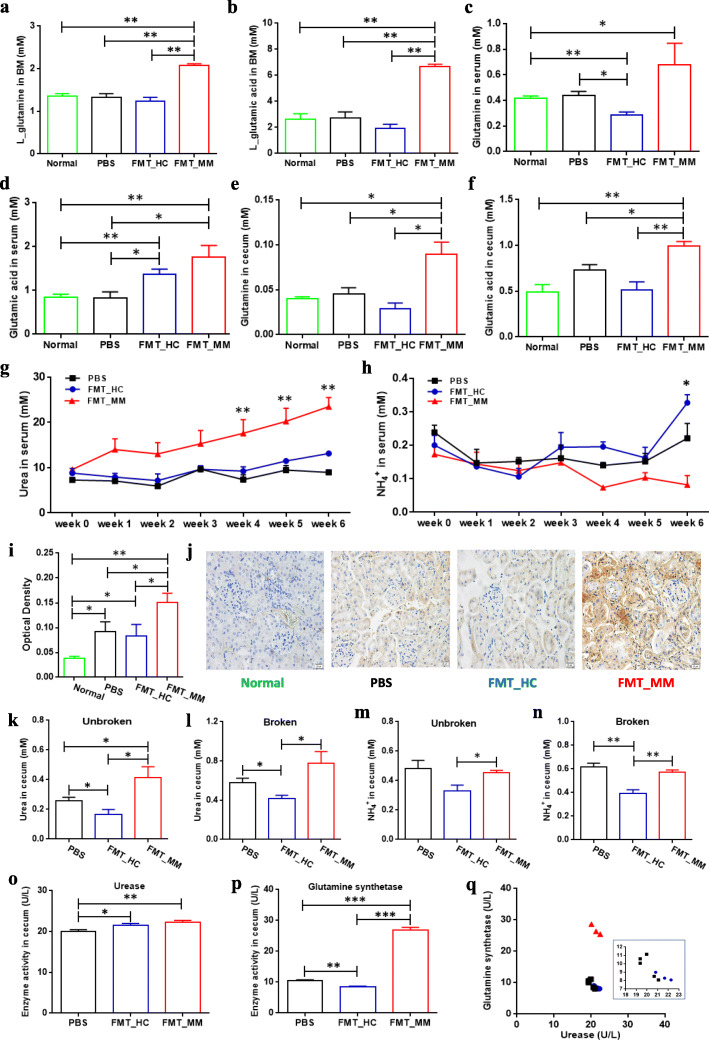
Differential metabolic profiling in FMT_HC, FMT_MM, and PBS mice. **a**, **b** The concentrations of BM l-glutamic acid (**a**) and l-glutamine (**b**) in FMT_HC, FMT_MM, PBS, and normal mice. *P* value was calculated by two-tailed unpaired *t* test. **c**, **d** The concentrations of serum l-glutamic acid (**c**) and l-glutamine (**d**) in FMT_HC, FMT_MM, PBS, and normal mice. *P* value was calculated by two-tailed unpaired *t* test. **e**, **f** The concentrations of cecal l-glutamic acid (**e**) and l-glutamine (**f**) in FMT_HC, FMT_MM, PBS, and normal mice. *P* value was calculated by two-tailed unpaired *t* test. **g**, **h** The concentrations of serum urea (**g**) and NH_4_^+^ (**h**) in FMT_HC, FMT_MM, and PBS mice, respectively. *P* value was determined by using two-way ANOVA test. **i**, **j** The deposition of monoclonal protein (lgG2b kappa) in the kidney surface of FMT_HC, FMT_MM, PBS, and normal mice. *P* value was determined by using one-tailed *t* test. **k**, **l** The concentrations of cecal urea after microbial cells being unbroken (**k**) and broken (**l**), respectively. *P* value was calculated using two-tailed unpaired *t* test. **m**, **n** The concentrations of cecal NH_4_^+^ after microbial cells being unbroken (**m**) and broken (**n**), respectively. *P* value was calculated using two-tailed unpaired *t* test. **o**, **p** The activities of cecal urease (**o**) and glutamine synthase (**p**) in FMT_HC, FMT_MM, and PBS mice, respectively. *P* value was calculated by using two-tailed unpaired *t* test. **q** Scatter plot of enzyme activities in cecal contents of FMT_HC, FMT_MM, and PBS mice. *P < 0.05, **P < 0.01, ***P < 0.001

To explore the potential causes of accelerating tumor progression in FMT_MM mice, we further investigated the important metabolites in the nitrogen cycling pathway of the mouse hosts at week 6. Primarily, by targeted metabolomics, several amino acids were found to be significantly higher in BM of FMT_MM mice such as l-tryptophan, suggesting the involvement of intestinal microflora in host metabolism (Figure S8, Additional file 11). Particularly, we detected that the concentrations of l-glutamic acid and l-glutamine in the BM of FMT_MM mice were dramatically higher than those in FMT_HC mice, PBS mice, and normal mice without MM induction (Fig. 6a, b). With one accord, high-level l-glutamic acid and l-glutamine were also observed in the serum of FMT_MM mice (Fig. 6c, d) and in the cecal contents of FMT_MM mice (Fig. 6e, f). Because all of the mice had the same diet, we hypothesized that the elevated l-glutamine levels result from de novo synthesis of the distinct colonized microbes in FMT_MM mice.

In addition, during the course of the experiment, we observed increased urea levels in the serum of all experimented mice including FMT_MM mice, FMT_HC mice, and PBS mice. However, the concentration of urea in the serum of FMT_MM mice increased more remarkably in comparison to those in FMT_HC and PBS mice (Fig. 6g). Interestingly, a reduced level of blood-NH_4_^+^ in FMT_MM mice was observed (Fig. 6h), possibly due to an increased synthesis of urea or monoclonal proteins or both. The gradual accumulation of urea was probably due to the gradual decline in renal function, because the experimental mice were found to exhibit more protein deposition of IgG2b kappa on the renal tubules than normal mice, suggesting compromised renal function in MM mice (Fig. 6i, j). Particularly, FMT_MM mice had the highest amount of IgG2b kappa, followed by FMT_HC mice and PBS mice. Moreover, we discovered that the concentration of urea in the cecal contents of FMT_MM mice was higher than that of FMT_HC mice and PBS mice regardless whether microbial cells were broken or unbroken (Fig. 6k, l). In contrast, the concentration of NH_4_^+^ was higher in FMT_MM mice than that in FMT_HC mice, while slightly decreasing in FMT_MM mice compared with the PBS mice regardless whether microbial cells were broken or unbroken (Fig. 6m, n). That might result from the consumption of NH_4_^+^ by de novo synthesis of amino acids in FMT_MM mice. And, URE and GS activities were markedly higher in the cecal contents of FMT_MM mice than that of PBS mice (Fig. 6o, p), while the GS to URE was close to that found in the feces of MM patients (Fig. 6q). These findings implied that the high-level l-glutamine in FMT_MM mice was the result of biosynthesis by MM-enriched bacteria. In this scenario, the MM-enriched bacteria may enhance the supply of l-glutamine by recycling urea nitrogen, resulting in subsequent facilitation of 5TGM1 cell proliferation accompanied by accelerated MM progression in FMT_MM mice.

Indeed, we verified that the ability of microbiota to convert urea into glutamine was stronger in the gut of FMT_MM mice at week 0, without the induction of MM tumorigenesis (Additional file 12). Moreover, we also repeated the FMT experiments, in which fecal donors from two MM patients and two HCs were separately applied to female or male mice. We obtained the same results as in previous experiments (Additional file 13), suggesting that the results are reproducible.

### *Klebsiella pneumoniae* promote MM progression via de novo synthesis of glutamine in mice

**Fig. 7:**
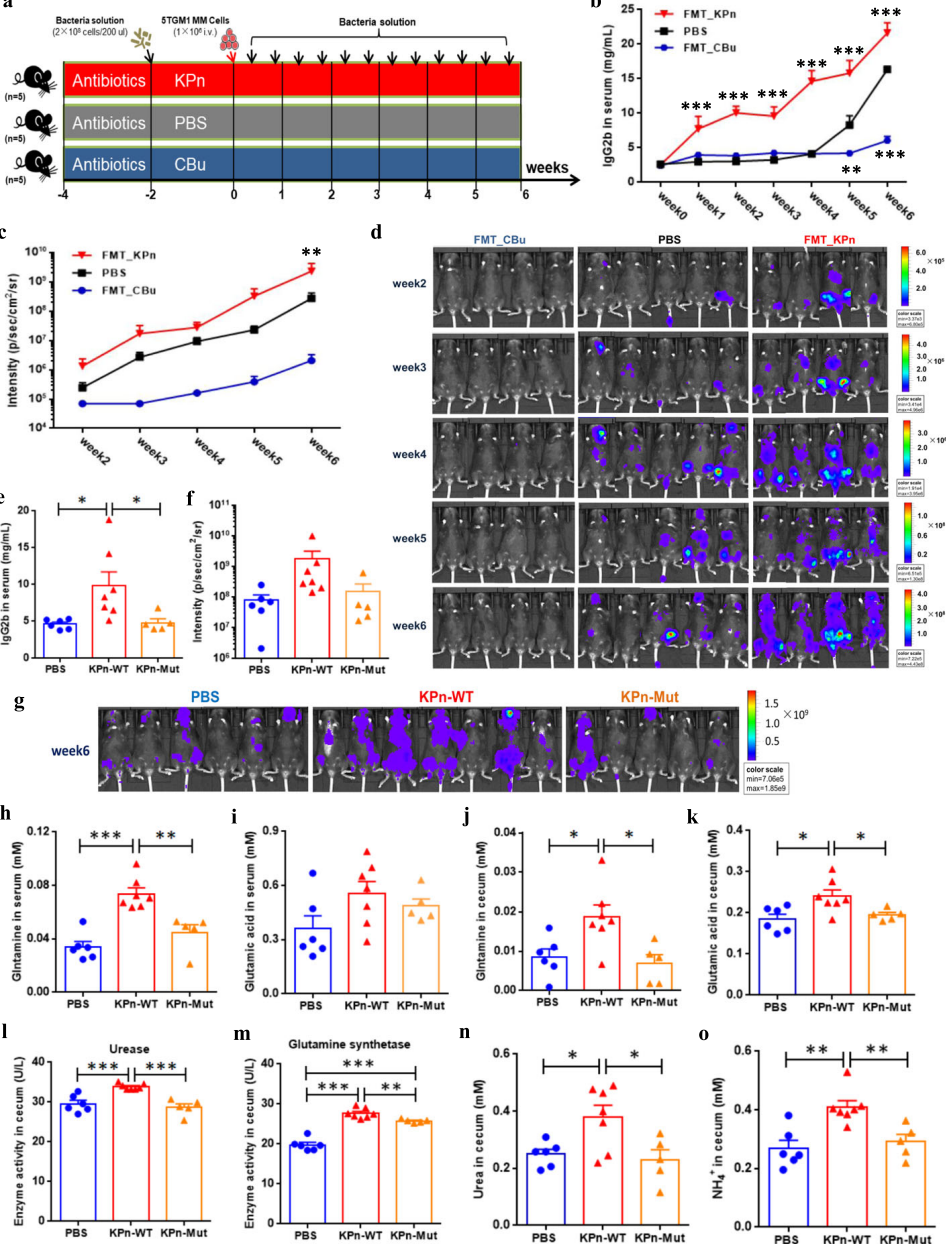
Mice experiment with *Klebsiella pneumoniae* and *Clostridium butyricum*. **a** Schematic representation of the characteristic bacteria transplantation experiment. **b**, **c** Lines in blue, in black, and in red denote the concentration of serum lgG2b (**b**) and the fluorescence intensity of live imaging (**c**) in FMT_CBu, PBS, and FMT_KPn mice, respectively. *P* value was determined by using two-way ANOVA test. **d** Live imaging of all recipient mice. **e**, **f** At week 6, the IgG2b concentration (**e**) and the tumor fluorescence intensity (**f**) in PBS, KPn_WT, and KPn_Mut mice, respectively. **g**, **h** At week 6, the concentrations of serum glutamine (**g**) and glutamic acid (**h**) in PBS, KPn_WT, and KPn_Mut mice, respectively. **i**, **j** At week 6, the concentrations of cecal glutamine (**i**) and glutamic acid (**j**) in PBS, KPn_WT, and KPn_Mut mice, respectively. **k**, **l** The activities of cecal urease (**k**) and glutamine synthase (**l**) in PBS, KPn_WT, and KPn_Mut mice, respectively. **m**, **n** The concentrations of cecal urea (**m**) and NH_4_^+^ (**n**) in PBS, KPn_WT, and KPn_Mut mice, respectively. *P* value was determined by using two-tailed unpaired *t* test. *P < 0.05, **P < 0.01, ***P < 0.001

To ask specifically whether the characteristic bacteria with differential abundance could impact the progression of MM, we performed FMT experiments using two bacteria with opposite effects—*Klebsiella pneumoniae* and *Clostridium butyricum*. We transplanted bacteria by gavage twice a week to amplify the effect on experiments (Fig. 7a). As expected, the IgG2b concentrations in FMT_KPn mice were significantly higher than that in PBS mice from week 1 to week 6, while the IgG2b concentration in FMT_CBu mice was substantially lower than that in PBS mice at week 5 and week 6 (Fig. 7b). Also, the same tendency was observed for tumor fluorescence intensity detected by live imaging of whole mice (Fig. 7c, d). Thus, the increase in *Klebsiella pneumoniae* abundance promoted MM progression, while the increase in *Clostridium butyricum* abundance mitigated MM progression in vivo.

To further examine whether *Klebsiella pneumoniae* promoted MM progression via glutamine synthesis, we conducted similar experiments using *Klebsiella pneumoniae* containing mutant *glnA* (in three groups: PBS, KPn_WT, KPn_Mut). At week 6, we detected heavier tumor burden in KPn_WT mice than that in KPn_Mut and PBS mice, including the concentration of IgG2b (Fig. 7e) and the tumor fluorescence intensity (Fig. 7f, g). In addition, as expected, higher levels of glutamine and glutamic acid were also detected in the serum (Fig. 7h, i) and cecal contents (Fig. 7j, k) of KPn_WT mice. Furthermore, in the cecal contents of KPn_WT mice, we discovered higher activities of URE and GS (Fig. 7l, m) and higher concentrations of urea (Fig. 7n) and NH_4_^+^ (Fig. 7o). Thus, wild-type *Klebsiella pneumoniae* promote MM progression via de novo synthesis of glutamine.

### The supply of glutamine promoted MM progression in mice

**Fig. 8:**
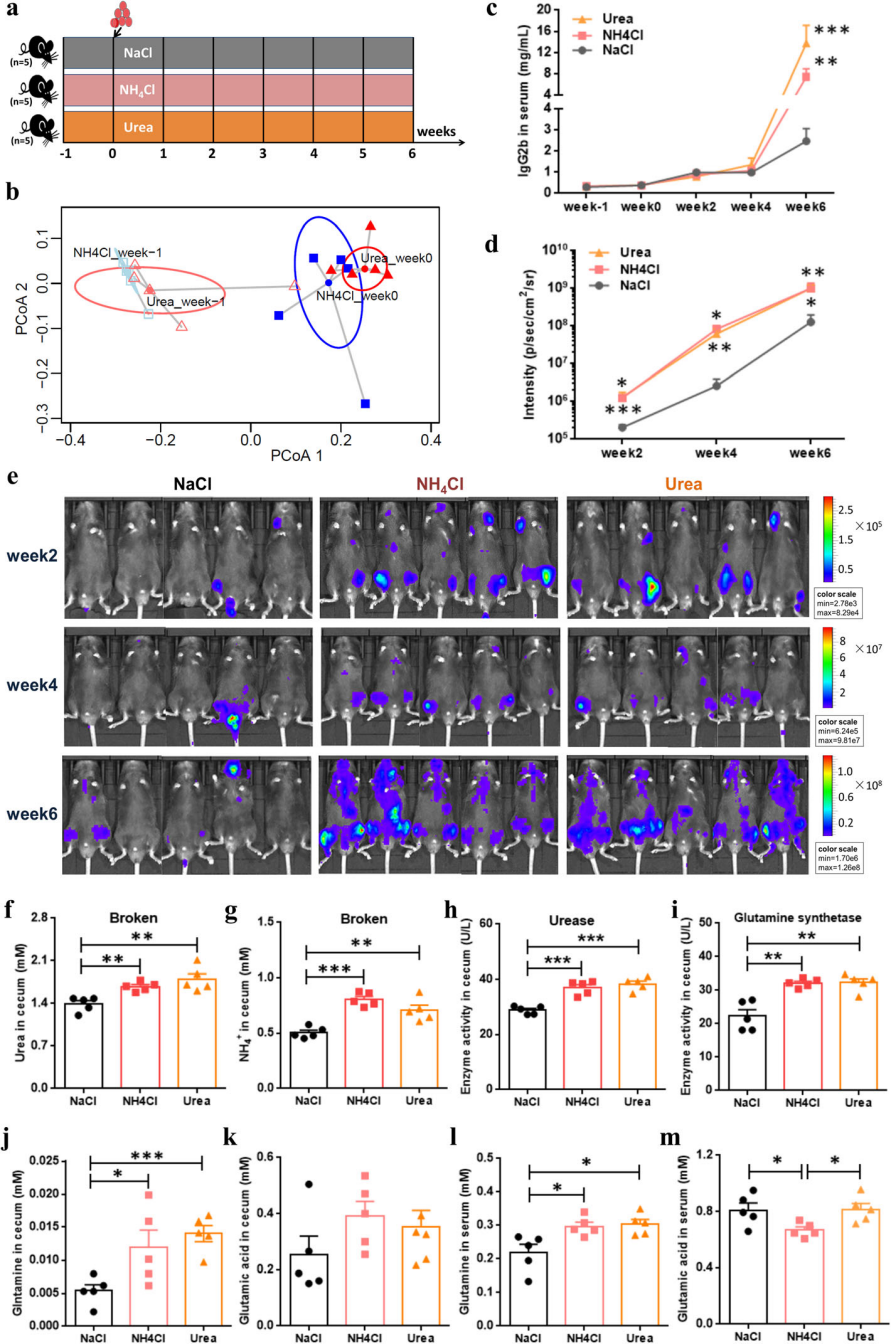
Mice experiment by gavage with ammonium and urea. **a** Schematic representation of the mice experiments. **b** Based on the Euclidean distance calculated from the relative abundances of several MM-enriched bacteria, experiments with PCoA show that these bacteria changed in NH_4_Cl and urea mice between week -1 and week 0. **c**, **d** Lines in gray, in pink, and in orange denote the serum lgG2b concentrations (**c**) and the tumor fluorescence intensity (**d**) in NaCl, NH_4_Cl, and urea mice, respectively. **e** Live imaging of all recipient mice. **f**, **g** At week 6, the concentrations of serum urea (**f**) and NH_4_^+^ (**g**) in NaCl, NH_4_Cl, and urea mice, respectively. **h**, **i** At week 6, the activities of cecal urease (**h**) and glutamine synthase (**i**) in NaCl, NH_4_Cl, and Urea mice, respectively. **j**, **k** At week 6, the concentrations of cecal glutamine (**j**) and glutamic acid (**k**) in NaCl, NH_4_Cl, and urea mice, respectively. **l**, **m** At week 6, the concentrations of serum glutamine (**l**) and glutamic acid (**m**) in NaCl, NH_4_Cl, and Urea mice, respectively. *P* value was determined by using two-tailed unpaired *t* test. *P < 0.05, **P < 0.01, ***P < 0.001

To ask whether the supply or the absence of glutamine could influence MM progression in mice, we performed two additional experiments. First, the mice were given by gavage with exogenous NH_4_^+^ or urea, with NaCl as the control (Fig. 8a). Subsequently, the Euclidean distance calculated from the relative abundances of several nitrogen-recycling bacteria as revealed by PCoA suggested that these bacteria changed in mice before and after treatment by gavage (Fig. 8b). Notably, we found that the mice treated with NH_4_^+^ or urea all showed faster MM progression, as monitored by the tumor fluorescence intensity from week 2 to week 6 (Fig. 8d, e). In addition, a much higher concentration of lgG2b was also detected at week 6 (Fig. 8c). However, no statistical difference was found when the mice treated with NH_4_^+^ or urea were compared. Furthermore, as expected, in the cecal contents of those mice treated with NH_4_^+^ or urea, there were higher concentrations of urea and NH_4_^+^ than controls (Fig. 8f, g). Accordingly, higher URE and GS activities were also discovered, suggesting the enrichment of urea nitrogen-recycling bacteria (Fig. 8h, i). As shown in Figure S9a (Additional file 14), most of the bacteria we tested showed higher relative abundance at week 0 and week 2, while subtle differences were found at week 4 and week 6, possibly due to the influence of MM progression in mice. Notably, we found that the mice treated with NH_4_^+^ or urea all showed significantly higher concentrations of glutamine in both the serum and cecal contents (Fig. 8j, l), although glutamate did not show a consistent trend when compared with controls (Fig. 8k, m). These results implied that the gut microbiota in mice can use NH_4_^+^ or urea for de novo synthesis of glutamine, thus promoting MM procession in mice.

**Fig. 9:**
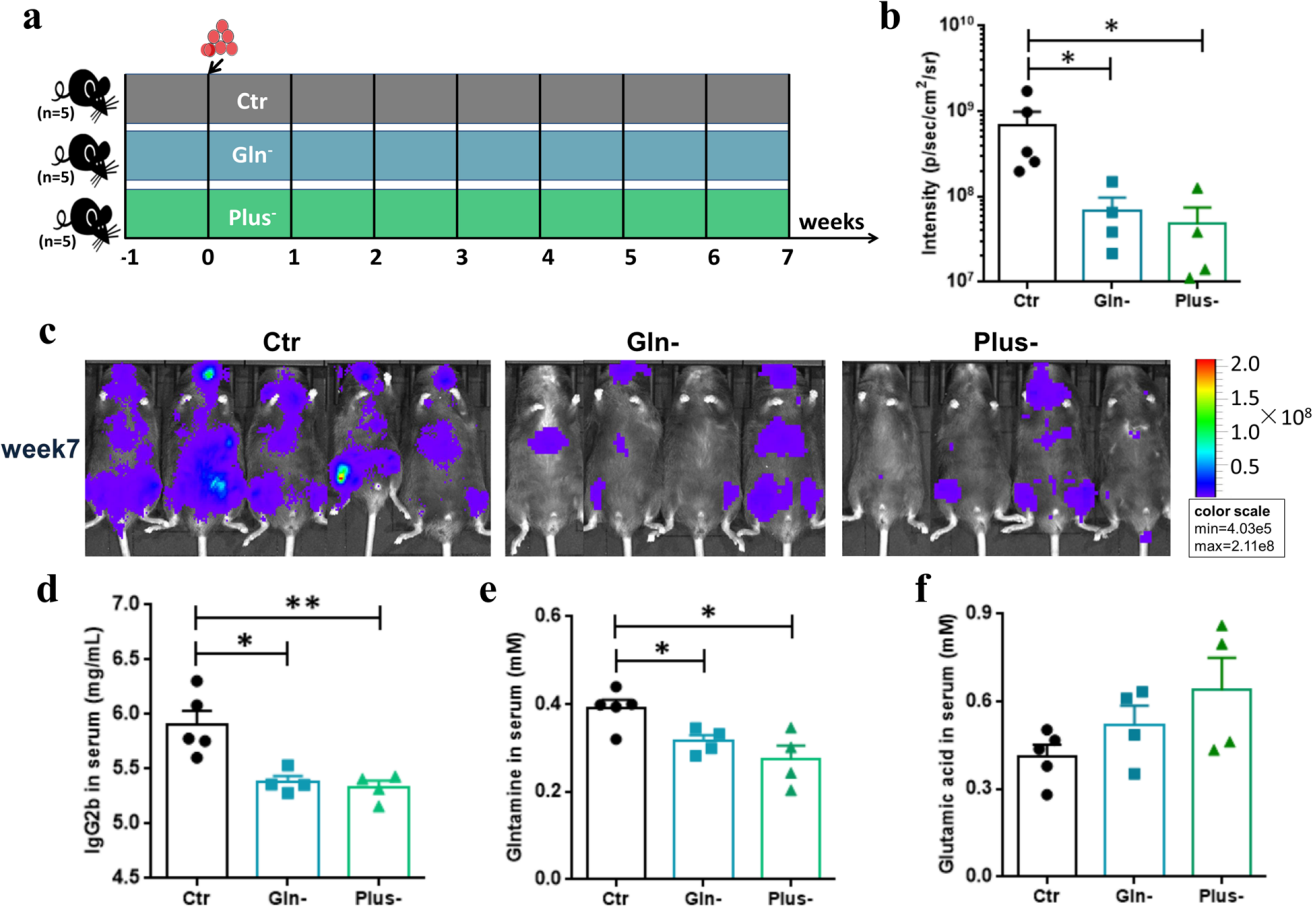
Mice experiment fed with glutamine- and cysteine-deficient diet. **a** Schematic representation of the mice experiment. **b** At week 7, the tumor fluorescence intensity in Ctr, Gln-, and Plus- mice, respectively. **c** Live imaging of all recipient mice at week 7. **d**–**f** The concentrations of serum lgG2b (**d**), glutamine (**e**), and glutamic acid (**f**) at week 7. *P* value was determined by using two-tailed unpaired *t* test. *P < 0.05, **P < 0.01, ***P < 0.001

In addition, we fed mice with glutamine- or/and cysteine-deficient diets to further examine the development of MM in mice (Fig. 9a). We found no significant difference between glutamine-deficient mice (Gln-) and glutamine/cysteine-deficient mice (Plus-) in MM progression. However, when compared with controls (Ctr), the treated mice (Gln-, Plus-) showed slower MM progression at week 7, as evidenced by the tumor fluorescence intensity (Fig. 9b, c) and the lgG2b concentration (Fig. 9d). As expected, much lower concentration of glutamine and higher concentration of glutamic acid were detected in the serum of the treated mice (Fig. 9e, f). Thus, the additional absence of glutamine in the diet may slow down the development of MM in mice.

## Discussion

In this study, the analysis of the gut microbiome of newly diagnosed MM patients in parallel with HCs at the metagenomic level led us to discover, for the first time, the significant differences in bacterial composition in MM patients. Specifically, several nitrogen-recycling bacteria such as *Klebsiella* and *Streptococcus* are enriched in MM subjects, and the enrichment is presumably driven by host-secreted nitrogen such as urea.

In addition, MM cells are known to be addicted to l-glutamine, leading to an accumulation of NH_4_^+^ in the BM and subsequent release into the circulation [35]. Normally, high-level blood-NH_4_^+^ can be converted to urea in the liver that is subsequently eliminated by the kidneys in urine [41, 42]. Excessive blood-NH_4_^+^ increases the incidence of hyperammonemic encephalopathy, a rare complication of MM with high mortality [43]. However, the deposition of monoclonal proteins during MM progression reduces renal function and impedes urea excretion [44]. Clinically, the levels of creatinine, urea, and uric acid in serum increase significantly from ISS stages I to III, while the level of blood-NH_4_^+^ shows a wide range [43, 45, 46]. Notably, in this study, several identified MM-enriched species reportedly hydrolyze urea or uric acid in the gastrointestinal tract, such as *Streptococcus* sp. and *Klebsiella* sp. [28]. And, the *Citrobacter*, *Enterobacter*, and *Klebsiella* all exhibited low correlations with other top genera in MM, suggesting that they were susceptible to interference from the surrounding environment (Figure S3, Additional file 3). Overall, our findings implied that excessive accumulation of nitrogen in the serum such as urea may enter the intestine and regulate microbial composition during MM progression. That was indirectly verified in a previous study on subjects with end-stage renal disease (ESRD), in which Wong et al. described the significant expansion of intestinal bacteria that contain urease, uricase, and indole and p-cresol-forming enzymes, and contraction of the bacteria producing short-chain fatty acids [47, 48]. We boldly speculate that the alterations in gut microbiome may derive from not only urea but also other substances accumulated as a result of kidney damage—e.g., uric acid.

**Fig. 10:**
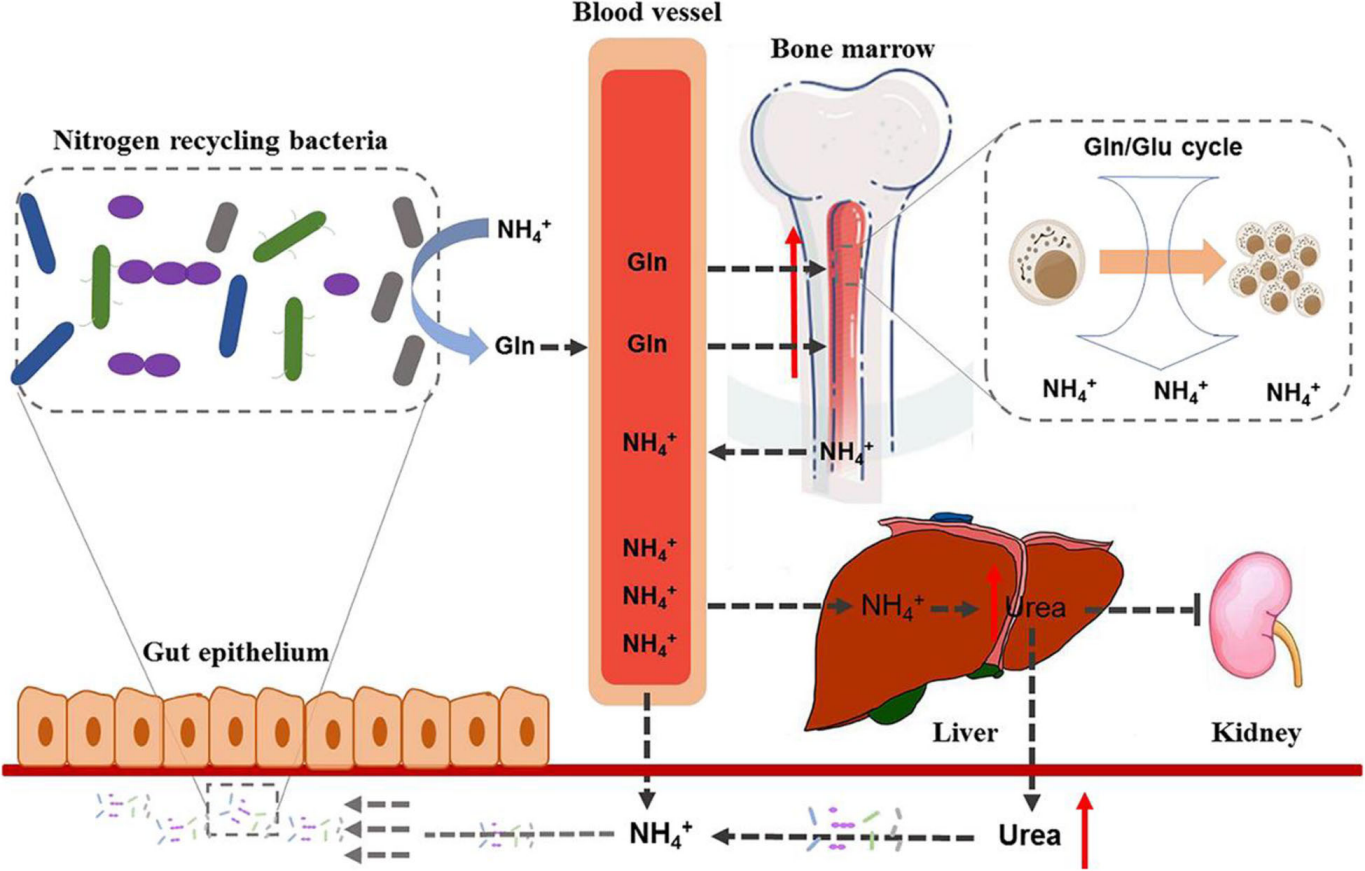
Schematic representation of the host-microbes interaction centered on nitrogen recycling

We reported, for the first time, that the gut microbiome in patients with MM played an active role in the malignant progression of MM. Our findings lead us to propose the following mechanism (Fig. 10), in which the accumulated urea or NH_4_^+^ and the descending renal function during MM progression would alter the intestinal bacterial composition, resulting in the preferential proliferation of nitrogen-recycling bacteria. As such, urea is efficiently hydrolyzed and used to synthesize l-glutamine that is delivered to the host, thereby accelerating the development of MM. Meanwhile, we consider that the altered gut microbiome is conducive to nitrogen recycling and utilization by the host-microbe superorganism.

Our findings also suggested that the alterations of the gut microbiome should be taken into consideration during MM treatment. Although most MM-enriched bacteria identified in this cohort are components of the normal intestinal flora, there are also opportunistic pathogens that could cause serious infections especially in high-risk groups of patients such as those with compromised immune systems or other underlying diseases (e.g., cancer). In particular, two of the identified MM-enriched bacteria—i.e., *Streptococcus pneumoniae* and *Klebsiella pneumoniae*—are responsible for the occurrence of pneumonia in patients with MM [27]. However, whether inhibition of MM-enriched bacteria can reduce the risk of infections in MM patients remains to be determined. Nevertheless, it was also found that the bacteria which produce short-chain fatty acids were depleted in MM and that the increase of *Clostridium butyricum* in MM mice mitigated tumor progression. These findings should open new avenues for MM treatment and evaluation.

## Conclusions

Taken together, our findings present the following mechanism: the alterations of the gut microbiome in MM patients result from the excessive accumulation of blood urea, and the altered gut microbiome in turn plays an active role in malignant progression of MM. We revealed the microbe-host interactions on the basis of urea nitrogen recycling and utilization in MM. Our discoveries open new avenues for novel treatment strategies via manipulation of the intestinal microbiota of MM patients. Also, these findings imply a broad mechanism that the elevated level of urea leads to preferential accumulation of ureolytic bacteria, which is conducive to nutrient conservation by the host-microbe superorganism.

## Materials and methods

### Participants and their sample collection in the cohort

In this study, we first collected feces samples from MM patients at the time of initial diagnosis without any clinical treatment. All patients had similar ages and BMI and were without gastrointestinal diseases. The sampling procedure was approved by the Cancer Research Institute of the Central South University Medical Ethics Committee. Here, 19 newly diagnosed MM patients including 14 males and 5 females were selected. Their average age and BMI index were 60.4 ± 6.2 and 22.9 ± 4.2, respectively. To match the numbers, 18 healthy controls were selected with the age of 57.9 ± 4.4 and BMI index of 24.3 ± 2.1, and the number of males and females was 13 and 5, respectively. Meanwhile, the age and BMI index of 19 patients and 18 controls were verified, and no significant difference was found (two-tailed Welch’s *t* test, *P* = 0.49). Similarly, the gender distribution of the two groups was also validated (Fisher’s exact test, *P* = 1) (Table S4a, Additional file 15).

Fresh fecal samples from each participant were collected and frozen in liquid nitrogen. Subsequently, samples were stored at – 80 °C until the time of transport on dry ice to Beijing Genomics Institute Genomics Co., Ltd for metagenomic sequencing. In the meantime, a fasting serum sample from each participant was also obtained. All samples were stored at – 80 °C until submission to Metabo-Profile Biotechnology (Shanghai) Co., Ltd for untargeted metabolomics detection.

Moreover, as described above, additional feces samples were also collected from 17 MM patients and 21 healthy controls (Table S4b, Additional file 15), which along with the metagenome sequencing samples was treated as an expanded cohort for the qPCR experiment. And, two serum samples from MGUS patients were also collected in this study (Table S4c, Additional file 15).

### Metagenomic sequencing, taxonomic classification, and functional annotation

The total DNA of each fecal sample was extracted using E.Z.N.A Stool DNA extraction kit (Omega Bio-tek, Norcross, Ga.) following the manufacturer’s instructions. The quality of DNA was analyzed using Qubit (Invitrogen, USA) and 1% agarose gel electrophoresis. All samples were sequenced on the Illumina platform (paired-end; insert size, 350 bp; read length, 150 bp). Raw sequences were processed to remove low-quality sequences using Trimmomatic (version 0.36), and human sequences were filtered out with the human reference genome (hg38) using Bowtie2 (version 2.2.4). The high-quality reads for the 37 samples were acquired, with an average of 25 Gb per sample.

Subsequently, the remaining high-quality reads were used for taxonomic classification using Kraken (version 1.0) and the standard Kraken database with default settings [14]. Although all samples were sequenced with a similar depth, the read counts table of several levels (e.g., phylum, class, order, family, genus, species) were rarefied to the minimum read counts to reduce the effects of uneven sampling in the cohort.

In addition, Metagenome Composition Vector (version 2.3.0) was used for functional annotation based on the clean sequences [29]. In this study, our study was focused mainly on alterations of functional proteins, and the KEGG level 4 (KEGG ontology) was therefore rarefied accordingly for subsequent identification of different functional proteins.

### Bacterial diversity in the cohort

To evaluate the sequencing depth and species richness in all subjects of the cohort, rarefaction analysis was performed by using R (version 3.5.0) and package vegan (version 2.5-2). To estimate the bacterial diversity, the Shannon–Weaver index at both species and genus level was also calculated by using R and package vegan.

In addition, the Bray-Curtis dissimilarity indices between samples at the species level were calculated by using R and package vegan, after the species read counts were subjected to square root transformation and Wisconsin double standardization. To estimate the between-sample (β) diversity, permutational multivariate analysis of variance (PERMANOVA) was performed based on the Bray-Curtis distances. Meanwhile, principal coordinate analysis (PCoA) was performed based on the Bray-Curtis dissimilarity matrix to visualize β diversity. Equally, the β diversity between samples at the KOs level was executed accordingly.

### Statistical analysis

On the R platform, metagenomic features (i.e., taxonomic and functional features) were analyzed statistically. The top four phyla and the top thirty genera were found to account for the majority of microbes, respectively, at the phylum and genus level. Their significance in MM and HC groups was determined by the *P* value of Wilcoxon rank sum test < 0.05. In addition, to focus on the more significant microbes, the species with low read-count percentages were considered as noise and removed from the matrix (threshold was set at 0.01%), and the species that were present in less than half of the subjects in the cohort were deleted. Similarly, the KOs that were present in less than half of the subjects in the cohort were excluded, and the threshold percentage of KOs was set at 0.001%. Significantly different species and KOs were all identified by R platform and using package DESeq2 (version 1.26.0) based on read counts [17], and the difference that was significant was determined with the absolute value of log base 2 of fold change > 1 and adjusted *P* value < 0.05. The different KOs were mapped to the corresponding modules based on KO database (https://www.kegg.jp/kegg/ko.html) [49]. Modules mapped by at least 2 KOs were presented. The Spearman’s correlation in this study was calculated by using R and package psych (version 1.8.12) and visualized by package pheatmap (version 1.0.12) or Cytoscape (version 3.4.0).

### Redundancy analysis

RDA was performed by using R (version 3.4.4) and package vegan (version 2.5-4), and the interactions were visualized by using package ggplot2 (version 3.1.1).

### 16S ribosomal DNA qPCR

The relative abundances of the different species identified by metagenomic sequencing were further tested using qPCR in both original fecal samples and more recently accumulated samples, which were collected from newly diagnosed patients with MM. The paired primers specific for each species were designed using Primerblast based on species-specific regions on the 16S ribosomal DNA (V1 or V2), while the conserved sequences were used for amplification of total bacteria [50]. Meanwhile, the coverage and specificity of each paired primers were also evaluated using an online tool TestPrime 1.0 (https://www.arb-silva.de). Primers are listed in Table S5 (Additional file 16), with the exception of bacteria sp. or complex and three species (i.e., *Butyrivibrio proteoclasticus*, *Clostridium botulinum*, *Prevotella fusca*), for which specific primers could not be designed. Besides, all primers were validated using gradient PCR to detect the annealing temperature and the specificity of primers.

Appropriate fecal samples were used to extract total bacterial DNAs (Mogen stool DNA kit), whose concentrations were subsequently measured using Nanodrop. One percent AGAR gel electrophoresis was used to detect whether the extracted DNA had been degraded (100 V 30 min). The reaction mixture (20 μl) for qPCR contained Applied Biosystems^TM^ PowerUp^TM^ SYBR^TM^ Green Master Mix, forward and reverse primer (final concentration 400 nM), and the extracted DNA (10 μl). The thermocycling program was 40 cycles and consisted of 95 °C for 15 s and 56 °C for 15 s and 72 °C for 1 min with an initial cycle of 50 °C for 2 min and 95 °C for 2 min. The melting curve was constructed in the range of 60 to 95 °C after PCR was performed.

Assuming that for all templates and primers a cycle equally doubles the number of template DNA, the relative abundance of a certain strain (*i*) can be calculated as follows:

$$ \mathrm{Relative}\ \mathrm{abundance}\ (i)=\frac{{\left(\frac{1}{2}\right)}^{\mathrm{CT}i}}{{\left(\frac{1}{2}\right)}^{\mathrm{CT}c}}={\left(\frac{1}{2}\right)}^{\mathrm{CT}i-\mathrm{CT}c}={\left(\frac{1}{2}\right)}^{\triangle \mathrm{CT}} $$

The cycle threshold of strain *i* primer and common primer (total bacteria) are represented by CT*i* and CT*c*, while △CT denotes the difference between them. From the equation, the logarithm of relative abundance negatively correlates linearly with △CT.

### Metabolomics analysis of human serum samples and mouse bone marrow samples

Gas chromatography coupled to time-of-flight mass spectrometry (GC-TOFMS) system (Pegasus HT, Leco Corp., St. Joseph, MO, USA) was used to quantify the detected metabolites. Metabolites were annotated using the mammalian metabolite database *JiaLib*^*TM*^ and employing a strict matching algorithm incorporated in the XploreMET software that uses both retention times and fragmentation patterns in the mass spectrum.

In human serum samples, a total of 180 measurable and reproducible metabolite signals were detected, 67 metabolites were unidentified. We therefore analyzed 113 identified metabolites and 28 ratios of metabolites from KEGG metabolic pathways (Table S6, Additional file 17). Statistical analysis of metabolic profiling of subjects was performed using the SIMCA software (SIMCA 14.1). Based on the results of metabolic profiling with Pareto scaling, the sophisticated multivariate statistical model, orthogonal partial least-squares discriminant analysis (OPLS-DA), was established to visualize differences in metabolite profiles. Meanwhile, the R2Y and Q2 of OPLS-DA model were 0.853 and 0.679, respectively. Metabolite signals causing the significant differences in metabolic profiling between two groups were screened on the basis of variable influence on projection (VIP) > 1 and *P* value of Welch’s *t* test < 0.05.

Mouse BM samples were prepared based on the method previously published with minor modifications [51–53]. All of the standards used were obtained from Sigma-Aldrich (St. Louis, MO, USA), Steraloids Inc. (Newport, RI, USA), and TRC Chemicals (Toronto, ON, Canada). All the standards were accurately weighed and prepared in an appropriate solution to obtain the individual stock solution at a concentration of 5.0 mg/mL. An appropriate amount of each stock solution was mixed to create stock calibration solutions. The raw data files generated by UPLC-MS/MS were processed using the TMBQ software (v1.0, Human Metabolomics Institute, Shenzhen, Guangdong, China) to perform peak integration, calibration, and quantitation for each metabolite. The current TMBQ is hosted on Dell PowerEdge R540 Servers operated with RedHat Enterprise Linux 7.5. The secured Java UI (User Interface) permits the user to have access to use a great variety of statistical tools for viewing and exploring project data on its own desire.

### The 5TGM1 mouse model

C57BL/KaLwRij mice of 6 weeks old were purchased from Harlan Laboratories Inc. (Harlan Mice, Netherlands). C57BL/KaLwRij mice can occasionally develop MM disease late in life, in which 5TGM1 cell line can be isolated. However, after 5TGM1 cells were injected into young mice, the cells colonized the bone marrow and caused myeloma. In this study, all animal studies were approved by the Ethical Committee for Animal Experiments of Central South University. The 8- to10-weeks-old mice were injected with the phosphate-buffered saline (PBS) vehicle or 8 × 10^5^ 5TGM1-luc cells, and the clinical endpoint was achieved when mice exhibited signs of hind limb weakness.

### Fecal microbiota transplantation experiment, sample collection, and detection

As indicated in Fig. 3 i, fresh stool from HC and MM patient was separately collected, and then stool (2.5 g per person) was mixed and suspended using PBS (10 mL). The suspension liquid was then filtered using 70 μm strainers, and the filtrate was centrifuged at 2000 rpm for 10 min. Subsequently, after the removal of the supernatant, the remaining pellet was resuspended using 2 mL PBS. The mixture was used for fecal microbiota transplantation by gavage (200 μL per mouse).

Before gavage was performed, all mice were treated with a cocktail of broad-spectrum antibiotics including ampicillin (0.2 g/L), vancomycin (0.1 g/L), neomycin (0.2 g/L), and metronidazole (0.2 g/L) in the drinking water for 2 weeks [40]. Subsequently, FMT_HC mice and FMT_MM mice, respectively, were given fecal suspension for 2 weeks by gavage (twice per week). In contrast, the mice of the PBS group were given PBS by gavage for 2 weeks (twice per week). To evaluate the influence of fecal microbiota transplantation on experiment mice, the stool was collected from FMT_HC mice and FMT_MM mice at week -4 and week 0 (before and after gavage). The relative abundances of several characteristic bacteria were separately measured, and PCoA was performed to determine whether the colonization of distinct microflora was successful in FMT_MM and FMT_HC mice, including *Clostridium butyricum*, *Streptococcus mitis*, *Streptococcus gordonii*, *Streptococcus pneumoniae*, *Raoultella ornithinolytica*, *Citrobacter freundii*, *Enterobacter cloacae*, *Klebsiella aerogenes*, *Klebsiella pneumoniae*, and *Klebsiella variicola*. Notably, *Anaerostipes hadrus*, *Clostridium saccharobutylicum*, *Streptococcus salivarius*, and *Streptococcus oralis* were undetectable in most of the mice.

At week 0, all mice were induced to develop MM via tail-vein injection of 5TGM1 cells (1 × 10^6^ cfu). Subsequently, serum sample from all mice was collected every week for the measurement of the concentration of lgG2b, NH_4_^+^, and urea; fecal sample from all mice was collected to measure the relative abundances of the species of interest. Live imaging of tumors was performed for all mice once per week from week 2. At week 6, after all mice were euthanized, cecum contents were collected for the measurement of the concentrations of l-glutamic acid, l-glutamine, NH_4_^+^, and urea, and the activities of urease and glutamine synthase [54]. In the meantime, the BMs from all mice were separately harvested from their femur and tibia by centrifugation at 4 °C (6000 rpm, 5 min). The cell-free supernatants of BM were prepared for targeted metabolomic detection. And the kidney tissue from all mice was separately collected for immunocytochemistry analysis.

ELISA was performed for the detection of mouse IgG2b in serum using Mouse IgG2b ELISA Quantification set (Bethyl Laboratories, Inc., USA). The concentration of NH_4_^+^ and urea in the serum was detected using the Blood Ammonia Assay Kit (Nanjing Jiancheng Biotechnology Co., Ltd., China) and Urea Detection Kit (diacetyl-monoxime colorimetric method) (Shanghai Zhuocai Biotechnology Co., Ltd., China), respectively. And the concentration of l-glutamic acid and l-glutamine was measured using the Glutamine Colorimetric Assay Kit (BioVision, Inc. USA).

In addition, the cecal contents (0.1 g) were dissolved using PBS (900 μL), and then shaken with glass beads (0.2 g, 425–600 μm) to break up the microorganisms inside (3000 rpm, 6 min). The mixture was centrifuged at 4 °C (5000 rpm, 6 min) to obtain supernatant for the subsequent measurement using the corresponding kits, including the concentration of l-glutamic acid, l-glutamine, urea, and ammonia and the activities of urease and glutamine synthetase. As shown in the kit instructions, before the measurement of l-glutamine, it was necessary to remove macromolecular proteins from samples by centrifuging in an ultrafiltration tube (14,000 rcf, 20 min) (UFC500396, Millipore, USA). The activities of urease and glutamine synthase were detected using Microorganism URE ELISA Kit (Shanghai Zhuocai biotechnology Co., Ltd., China) and Microorganism GS ELISA Kit (Shanghai Zhuocai biotechnology Co., Ltd., China), respectively. In the statistical analysis of the results, we excluded the dead mice and the outliers.

### The characteristic bacteria transplantation experiment and sample collection

Experimental mice were also gavaged with 2 × 10^8^ cfu/200μL of *Klebsiella pneumoniae* (BNCC 102997 = ATCC 10031) and *Clostridium butyricum* (BNCC 337239 = ATCC 19398), which were purchased from Beijing BeiNa Biotechnology Institute. *Klebsiella pneumoniae* was cultured in nutrient broth (Product ID: 022010, Guangdong HuanKai Microbial Co., Ltd., China) at 37 °C and 200 rpm for ~ 16 h, while *Clostridium butyricum* was statically cultured in thioglycollate medium (Product ID: HB5191, Qingdao Hope Bio Technology Co., Ltd., China) under anaerobic conditions at 37 °C for ~ 18 h. In addition, as previously described [55], *Klebsiella pneumoniae* with mutant *glnA* (coding glutamine synthetase, EC: 6.3.1.2) was constructed by homologous recombination technique using plasmid pKO3-Km. According to the turbidity/absorbance of the fermentation broth, there was no significant difference in the growth of wild-type *K. pneumoniae* and mutants. Here, for the purpose, we selected Mut3 for subsequent experiments, due to less *glnA* expression and less remaining glutamine in broth (Figure S10, Additional file 18). Microorganism cells were obtained from fermentation broth and were resuspended with PBS for subsequent gavage. Each mouse was given by gavage 200 μL suspension (about 2 × 10^8^ cells).

Similarly, all experimental mice were treated with a broad-spectrum cocktail of antibiotics for 2 weeks, and then each group of mice was transplanted with a specific fresh fermentation broth for 2 weeks (twice per week). At week 0, all experimental mice were induced to develop MM via tail-vein injection of 5TGM1 cells (1 × 10^6^ cfu). To amplify the effect on experiments, microbial transplantation continued twice a week. Meanwhile, serum lgG2b and tumor fluorescence intensity from all mice were monitored every week.

### Gavage experiments with ammonium and urea

We carried out mouse studies with NH_4_Cl (100 mM) and urea (50 mM) by gavage, with the control being with NaCl (0.9%). Each group of mice (i.e., NaCl, NH_4_Cl, and Urea) was given 100 μL by gavage once a day. After 1 week of gavage, all mice were induced to develop MM via tail-vein injection of 5TGM1 cells (1 × 10^6^ cfu) at week 0 (Fig. 8a), and afterwards all mice still received gavage daily.

To see if gavage can change the abundance of bacteria, the stool was collected from all mice at week -1, week 0, week 2, week 4, and week 6. Subsequently, these stool samples were used to measure separately the relative abundances of several MM-enriched bacteria, including *Streptococcus mitis*, *Streptococcus gordonii*, *Streptococcus pneumoniae*, *Raoultella ornithinolytica*, *Citrobacter freundii*, *Enterobacter cloacae*, *Klebsiella aerogenes*, *Klebsiella pneumoniae*, and *Klebsiella variicola*. At week 6, after all mice were sacrificed, cecal contents were collected for the measurement of the concentrations of l-glutamic acid, l-glutamine, NH_4_^+^, and urea and the determination of the activities of urease and glutamine synthase. In addition, serum samples were collected to measure the concentrations of l-glutamic acid and l-glutamine.

### Mouse studies using defective diet and sample collection

We also conducted mouse experiments, in which mice were fed with glutamine-deficient diet (Gln-) or glutamine/cysteine-deficient diet (Plus-). The mice fed with a holistic diet (Ctr) were used as controls. After 1 week of feeding, all mice were induced to develop MM via tail-vein injection of 5TGM1 cells (1 × 10^6^ cfu) at week 0 (Fig. 9a), and their diet remained the same. During the experimental process, the MM progression of mice was monitored by the use of tumor fluorescence intensity and was found to be most significant at week 7. Thus, at week 7, serum samples were collected for the measurement of the concentrations of lgG2b, l-glutamic acid, and l-glutamine.

### Immunocytochemistry analysis

The collected kidney tissue samples from all the mice were first fixed with paraformaldehyde, embedded in paraffin after dehydration, and sliced for immunohistochemistry. The slides then were subjected to dewaxing, rehydration, and hydrogen peroxide treatment. Subsequently, the tissue sections were incubated with anti-IgG2b kappa antibody in a 1:2000 dilution overnight at 4 °C. Next, the slides were incubated with HRP-conjugated secondary antibody and stained with 3,3′-diaminobenzidine tetrahydrochloride hydrate (DAB) for 3 min. Finally, cell nuclei were counterstained with hematoxylin. The stained sections were evaluated by using PerkinElmer Quantitative Pathology Imaging System with software inForm 2.4.

## Supplementary information

**Additional file 1: Figure S1.** Statistical analysis of microbes at the phylum level in the cohort. (a) Barplot shows the four most abundant microbial phyla in HC and MM groups, which represent more than 99% of the gut microbiota. (b) Boxplot illustrates a comparison between the top four microbial phyla abundances, where the boxes in blue or red denote samples from HC or MM groups, respectively. The significance was determined by using adjusted P-value from two-tailed Wilcoxon rank-sum test. The boxes represent the interquartile ranges (IQRs) between the first and third quartiles, and the line inside the box shows the median; whiskers denote the lowest or highest values within 1.5 times IQR from the first or third quartiles. Circles represent data points beyond the whiskers. ▪ adj. *P* > 0.05, * adj. *P* < 0.05.

**Additional file 2: Figure S2.** Boxplot shows the thirty most abundant microbial genera, accounting for about 96% of gut microbiota. The boxes in blue or red denote samples from HC or MM groups, respectively. The significance was determined by P-value from the two-tailed Wilcoxon rank-sum test. Boxes represent the interquartile ranges (IQRs) between the first and third quartiles, and the line inside the box shows the median; whiskers denote the lowest or highest values within 1.5 times of IQR from the first or third quartiles. Circles represent data points beyond the whiskers. ▪ adj. *P* > 0.05, * adj. *P* < 0.05, ** adj. *P* < 0.01, *** adj. *P* < 0.001.

**Additional file 3: Figure S3.** Co-occurrence network derived from the Spearman’s correlation (Rho>0.5, *P*-value <0.05) between the top 30 genera in HC&MM subjects (a), HC subjects (b), and MM subjects (c). The edge in magenta or in cyan denotes the positive correlation coefficient or negative correlation coefficient, respectively, while the size of each edge reflects the weight of correlation. Nodes represent genus, the size of which reflects the node degree. The gray nodes represent the genera that are not significantly different between HC and MM groups, while the red nodes represent the MM-enriched genera (see **Figure S2**).

**Additional file 4: Table S1.** The strong Spearman’s correlation (Rho>0.5 & *P*<0.05) between the thirty most abundant genera was calculated in HC&MM samples (a), HC samples (b), and MM samples (c).

**Additional file 5: Table S2.** The differential species and subspecies identified by DESeq2 (|log_2_(Fold Change)|>1 & adjusted *P*<0.05). The species and subspecies in red are enriched in MM, while the species and subspecies in blue are enriched in HC. And, the species in bold were further examined using qPCR in an expanded cohort, due to a padj less than 0.01.

**Additional file 6: Figure S4.** Graphs show the species without statistical difference in an expanded cohort using qPCR. HC-enriched species are highlighted in the blue frame, while MM-enriched species in the red frame. In HC, the circles in red and white represent the subjects from a new collection of controls and metagenomic sequenced groups, respectively. In MM, the squares in red and white represent the subjects from a new collection of MM patients and metagenomic sequenced groups, respectively. *P*-value was determined by using two-tailed Mann-Whitney test. Note that some species were undetected in some samples. There are 1, 3, 18, 12, 1, 15, 5, 10, 3, 8, 8, 16, 6, 24, and 6 undetected samples for *Streptococcus anginosus*, *Streptococcus parasanguinis*, *Intestinmonas butyriciproducens*, *Prevotella ruminicola*, *Prevotella melaninogenica*, *Collinsella aerofaciens*, *Bifidobacterium dentium*, *Fusobacterium varium*, *Bifidobacterium catenulatum*, *Bifidobacterium kashiwanohense*, *Bifidobacterium pseudocatenulatum*, *Lachnoclostridium phytofermentans*, *Herbinix luporum*, *Clostridium beijerinckii*, and *Clostridium difficile*, respectively.

**Additional file 7: Figure S5.** The differential taxa in HC and MM subjects. (a) The circles in red and in blue represent MM-enriched and HC-enriched taxa, respectively. (b) The abundance of MM-enriched taxa in MM patients with ISS stage-II and ISS stage-III, respectively.

**Additional file 8: Table S3.** The differential KOs identified by DESeq2 [|log_2_(Fold Change)|>1 & adjusted *P*<0.05]. The KOs with log_2_FoldChange more than 1 suggest MM-enriched.

**Additional file 9: Figure S6.** Heatmap shows the Spearman’s correlation between the 36 differential species and 26 differential metabolites. *, +, and # all suggest the significance; * *P*<0.05, + *P*<0.01, # *P*<0.001

**Additional file 10: Figure S7.** PCoA was separately performed based on the Euclidean distance calculated from the relative abundances of several characteristic bacteria at week 0 (a), week 2 (b), week 4 (c), and week 6 (d).

**Additional file 11: Figure S8.** Graphs show the concentrations of targeted metabolites in the bone marrow of Normal mice, PBS mice, FMT_HC, and FMT_MM mice. *P*-value was calculated by two-tailed unpaired t-test. * *P*<0.05, ** *P*<0.01, *** *P*<0.001.

**Additional file 12:** The ability of microbiota to convert urea into glutamine was stronger in the gut of FMT_MM mice at week 0, without the induction of MM tumorigenesis.

**Additional file 13:** The FMT experiment was repeated twice, in which fecal donors from two MM patients and two HCs were separately applied to female or male mice. And the same results were detected as previous experiment, suggesting the experimental results are reproducible.

**Additional file 14: Figure S9.** The changes in relative abundances of several MM-enriched bacteria. (a) Heatmap reflects the changes of the scaled relative abundance of bacteria over time in NaCl, NH_4_Cl, and Urea mice. (b) PCoA was performed based on the Euclidean distance calculated from the relative abundances of bacteria at week 0, week 2, week 4, and week 6.

**Additional file 15: Table S4.** The subjects’ characteristics of metagenome sequencing samples in this study (a). The subjects’ characteristics of un-metagenome sequencing samples in the expanded cohort of this study (b). The characteristics of two MGUS patients in this study (c).

**Additional file 16: Table S5.** The list presents the primers used in this study.

**Additional file 17:Table S6.** Untargeted metabolomic characteristics of human serum sample in this study. Metabolites in yellow are the identified differential metabolites in this study (*P*_value <0.05 & VIP >1).

**Additional file 18: Figure S10.** Construction and verification of *glnA*-mutant *Klebsiella pneumoniae* by homologous recombination technique using plasmid pKO3-Km. (a) Schematic depiction of *glnA* sequencing, in which fragment in blue was deleted. (b) Schematic diagram of plasmid pKO3-Km. (c) Primers used for *glnA* disruption and *glnA* qPCR were designed according to the *Klebsiella pneumoniae* subsp. HS11286 chromosome sequence (NC_016845.1: c36644-35196) and plasmid pKO3-Km sequence. (d) Agarose gel for PCR products, the left panel shows that with *Klebsiella pneumoniae* genomic DNA as the template; the right panel shows that with the mix left PCR products as a template. (e) Agarose gel for PCR products of 10 clones of pKO3-km-glnAmut. (f) NCBI Blast sequence alignment for pKO3-km-glnAmut. (g) The relative abundance of gene *glnA* in the clones Mut1 to 10 and wild-type *Klebsiella pneumoniae* using qPCR. (h) The remaining concentrations of glutamine in the broth. Blk represents the initial concentration.

## Data Availability

Sequence files for all samples used in this study have been deposited in the public database of the National Omics Data Encyclopedia (NODE) under project number OEP000194, with the available url at https://www.biosino.org/node/review/detail/OEV000075?code=SEPGGE5F. All scripts are available in GitHub (https://github.com/XingxingJian/metagenome_MM_code).
