# Supplementary material for: Alterations of gut microbiome accelerate multiple myeloma progression by increasing the relative abundances of nitrogen-recycling bacteria
Source: Microbiome. 2020 May 28;8:74. doi: 10.1186/s40168-020-00854-5 (PMC7257554; doi:10.1186/s40168-020-00854-5)

**Additional file 1: Figure S1. Statistical analysis of microbes at the phylum level in the cohort**

**(a)** Barplot shows the four most abundant microbial phyla in HC and MM groups, which represent more than 99% of the gut microbiota. **(b)** Boxplot illustrates a comparison between the top four microbial phyla abundances, where the boxes in blue or red denote samples from HC or MM groups, respectively. The significance was determined by using adjusted *P*-value from two-tailed Wilcoxon rank-sum test. The boxes represent the interquartile ranges (IQRs) between the first and third quartiles, and the line inside the box shows the median; whiskers denote the lowest or highest values within 1.5 times IQR from the first or third quartiles. Circles represent data points beyond the whiskers. ▪ adj. *P* > 0.05, * adj. *P* < 0.05.


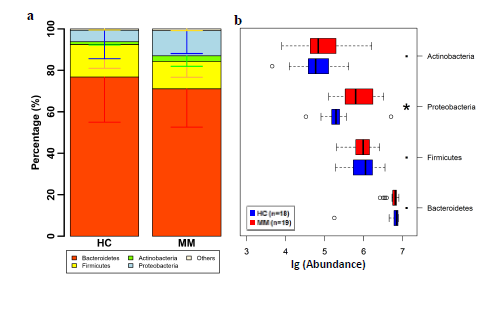

Supplement: Supplementary file 2 — Additional file 1: Figure S1. Statistical analysis of microbes at the phylum level in the cohort. (a) Barplot shows the four most abundant microbial phyla in HC and MM groups, which represent more than 99% of the gut microbiota. (b) Boxplot illustrates a comparison between the top four microbial phyla abundances, where the boxes in blue or red denote samples from HC or MM groups, respectively. The significance was determined by using adjusted P-value from two-tailed Wilcoxon rank-sum test. The boxes represent the interquartile ranges (IQRs) between the first and third quartiles, and the line inside the box shows the median; whiskers denote the lowest or highest values within 1.5 times IQR from the first or third quartiles. Circles represent data points beyond the whiskers. ▪ adj. P > 0.05, * adj. P < 0.05. [file 40168_2020_854_MOESM1_ESM.docx]
