# Supplementary material for: Alterations of gut microbiome accelerate multiple myeloma progression by increasing the relative abundances of nitrogen-recycling bacteria
Source: Microbiome. 2020 May 28;8:74. doi: 10.1186/s40168-020-00854-5 (PMC7257554; doi:10.1186/s40168-020-00854-5)

**Additional file 2: Figure S2. Boxplot shows the thirty most abundant microbial genera, accounting for about 96% of gut microbiota.** The boxes in blue or red denote samples from HC or MM groups, respectively. The significance was determined by *P*-value from the two-tailed Wilcoxon rank-sum test. Boxes represent the interquartile ranges (IQRs) between the first and third quartiles, and the line inside the box shows the median; whiskers denote the lowest or highest values within 1.5 times of IQR from the first or third quartiles. Circles represent data points beyond the whiskers. ▪ adj. *P* > 0.05, * adj. *P* < 0.05, ** adj. *P* < 0.01, *** adj. *P* < 0.001.


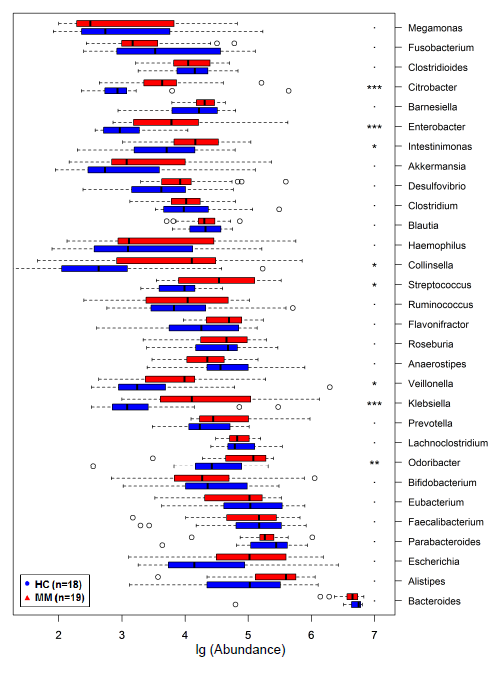

Supplement: Supplementary file 3 — Additional file 2: Figure S2. Boxplot shows the thirty most abundant microbial genera, accounting for about 96% of gut microbiota. The boxes in blue or red denote samples from HC or MM groups, respectively. The significance was determined by P-value from the two-tailed Wilcoxon rank-sum test. Boxes represent the interquartile ranges (IQRs) between the first and third quartiles, and the line inside the box shows the median; whiskers denote the lowest or highest values within 1.5 times of IQR from the first or third quartiles. Circles represent data points beyond the whiskers. ▪ adj. P > 0.05, * adj. P < 0.05, ** adj. P < 0.01, *** adj. P < 0.001. [file 40168_2020_854_MOESM2_ESM.docx]
