# Supplementary material for: Alterations of gut microbiome accelerate multiple myeloma progression by increasing the relative abundances of nitrogen-recycling bacteria
Source: Microbiome. 2020 May 28;8:74. doi: 10.1186/s40168-020-00854-5 (PMC7257554; doi:10.1186/s40168-020-00854-5)

**Additional file 3: Figure S3. Co-occurrence network derived from the Spearman’s correlation** (Rho>0.5, p-value <0.05) **between the top 30 genera in HC&MM subjects (a), HC subjects (b), and MM subjects (c)**. The edge in magenta or in cyan denotes the positive correlation coefficient or negative correlation coefficient, respectively, while the size of each edge reflects the weight of correlation. Nodes represent genus, the size of which reflects the node degree. The gray nodes represent the genera that are not significantly different between HC and MM groups, while the red nodes represent the MM-enriched genera (see **Figure S2**).


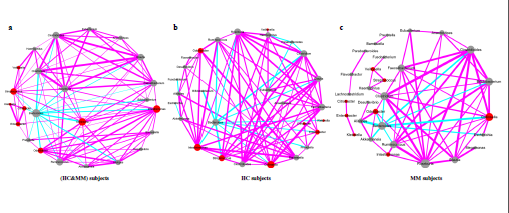

Supplement: Supplementary file 4 — Additional file 3: Figure S3. Co-occurrence network derived from the Spearman’s correlation (Rho>0.5, P-value <0.05) between the top 30 genera in HC&MM subjects (a), HC subjects (b), and MM subjects (c). The edge in magenta or in cyan denotes the positive correlation coefficient or negative correlation coefficient, respectively, while the size of each edge reflects the weight of correlation. Nodes represent genus, the size of which reflects the node degree. The gray nodes represent the genera that are not significantly different between HC and MM groups, while the red nodes represent the MM-enriched genera (see Figure S2). [file 40168_2020_854_MOESM3_ESM.docx]
