# Supplementary material for: Alterations of gut microbiome accelerate multiple myeloma progression by increasing the relative abundances of nitrogen-recycling bacteria
Source: Microbiome. 2020 May 28;8:74. doi: 10.1186/s40168-020-00854-5 (PMC7257554; doi:10.1186/s40168-020-00854-5)
Supplement: Supplementary file 7 — Additional file 6: Figure S4. Graphs show the species without statistical difference in an expanded cohort using qPCR. HC-enriched species are highlighted in the blue frame, while MM-enriched species in the red frame. In HC, the circles in red and white represent the subjects from a new collection of controls and metagenomic sequenced groups, respectively. In MM, the squares in red and white represent the subjects from a new collection of MM patients and metagenomic sequenced groups, respectively. P-value was determined by using two-tailed Mann-Whitney test. Note that some species were undetected in some samples. There are 1, 3, 18, 12, 1, 15, 5, 10, 3, 8, 8, 16, 6, 24, and 6 undetected samples for Streptococcus anginosus, Streptococcus parasanguinis, Intestinmonas butyriciproducens, Prevotella ruminicola, Prevotella melaninogenica, Collinsella aerofaciens, Bifidobacterium dentium, Fusobacterium varium, Bifidobacterium catenulatum, Bifidobacterium kashiwanohense, Bifidobacterium pseudocatenulatum, Lachnoclostridium phytofermentans, Herbinix luporum, Clostridium beijerinckii, and Clostridium difficile, respectively. [file 40168_2020_854_MOESM6_ESM.docx]

**Additional file 6: Figure S4 Graphs show the species without statistical difference in an expanded cohort using qPCR**. HC-enriched species are highlighted in the blue frame, while MM-enriched species in the red frame. In HC, the circles in red and white represent the subjects from a new collection of controls and metagenomic sequenced groups, respectively. In MM, the squares in red and white represent the subjects from a new collection of MM patients and metagenomic sequenced groups, respectively. *P*-value was determined by using two-tailed Mann-Whitney test. Note that some species were undetected in some samples. There are 1, 3, 18, 12, 1, 15, 5, 10, 3, 8, 8, 16, 6, 24, and 6 undetected samples for *Streptococcus anginosus*, *Streptococcus parasanguinis*, *Intestinmonas butyriciproducens*, *Prevotella ruminicola*, *Prevotella melaninogenica*, *Collinsella aerofaciens*, *Bifidobacterium dentium*, *Fusobacterium varium*, *Bifidobacterium catenulatum*, *Bifidobacterium kashiwanohense*, *Bifidobacterium pseudocatenulatum*, *Lachnoclostridium phytofermentans*, *Herbinix luporum*, *Clostridium beijerinckii*, and *Clostridium difficile*, respectively.
