# Supplementary material for: Alterations of gut microbiome accelerate multiple myeloma progression by increasing the relative abundances of nitrogen-recycling bacteria
Source: Microbiome. 2020 May 28;8:74. doi: 10.1186/s40168-020-00854-5 (PMC7257554; doi:10.1186/s40168-020-00854-5)
Supplement: Supplementary file 8 — Additional file 7: Figure S5. The differential taxa in HC and MM subjects. (a) The circles in red and in blue represent MM-enriched and HC-enriched taxa, respectively. (b) The abundance of MM-enriched taxa in MM patients with ISS stage-II and ISS stage-III, respectively. [file 40168_2020_854_MOESM7_ESM.docx]

**Additional file 7: Figure S5. The differential taxa in HC and MM subjects**

**(a)** The circles in red and in blue represent MM-enriched and HC-enriched taxa, respectively.

**(b)** The abundance of MM-enriched taxa in MM patients with ISS stage-Ⅱ and ISS stage-Ⅲ, respectively.
