# Supplementary material for: Alterations of gut microbiome accelerate multiple myeloma progression by increasing the relative abundances of nitrogen-recycling bacteria
Source: Microbiome. 2020 May 28;8:74. doi: 10.1186/s40168-020-00854-5 (PMC7257554; doi:10.1186/s40168-020-00854-5)
Supplement: Supplementary file 10 — Additional file 9: Figure S6. Heatmap shows the Spearman’s correlation between the 36 differential species and 26 differential metabolites. *, +, and # all suggest the significance; * P<0.05, + P<0.01, # P<0.001 [file 40168_2020_854_MOESM9_ESM.docx]

**Additional file 9: Figure S6. Heatmap shows the Spearman’s correlation between the 36 differential species and 26 differential metabolites**, in which the red and blue tags denote the enrichment in MM and HC samples, respectively. *, +, and # all suggest the significance; * *P*<0.05, + *P*<0.01, # *P*<0.001.
