# Supplementary material for: Alterations of gut microbiome accelerate multiple myeloma progression by increasing the relative abundances of nitrogen-recycling bacteria
Source: Microbiome. 2020 May 28;8:74. doi: 10.1186/s40168-020-00854-5 (PMC7257554; doi:10.1186/s40168-020-00854-5)
Supplement: Supplementary file 11 — Additional file 10: Figure S7. PCoA was separately performed based on the Euclidean distance calculated from the relative abundances of several characteristic bacteria at week 0 (a), week 2 (b), week 4 (c), and week 6 (d). [file 40168_2020_854_MOESM10_ESM.docx]

**Additional file 10: Figure S7. PCoA was separately performed based on the Euclidean distance calculated from the relative abundances of several characteristic bacteria at week 0 (a), week 2 (b), week 4 (c), and week 6 (d).**
