# Supplementary material for: Alterations of gut microbiome accelerate multiple myeloma progression by increasing the relative abundances of nitrogen-recycling bacteria
Source: Microbiome. 2020 May 28;8:74. doi: 10.1186/s40168-020-00854-5 (PMC7257554; doi:10.1186/s40168-020-00854-5)
Supplement: Supplementary file 12 — Additional file 11: Figure S8. Graphs show the concentrations of targeted metabolites in the bone marrow of Normal mice, PBS mice, FMT_HC, and FMT_MM mice. P-value was calculated by two-tailed unpaired t-test. * P<0.05, ** P<0.01, *** P<0.001. [file 40168_2020_854_MOESM11_ESM.docx]

**Additional file 11: Figure S8. Graphs show the concentrations of targeted metabolites in the bone marrow of Normal mice, PBS mice, FMT_HC, and FMT_MM mice**. *P*-value was calculated by two-tailed unpaired t-test. * *P*<0.05, ** *P*<0.01, *** *P*<0.001.
