# Supplementary material for: Alterations of gut microbiome accelerate multiple myeloma progression by increasing the relative abundances of nitrogen-recycling bacteria
Source: Microbiome. 2020 May 28;8:74. doi: 10.1186/s40168-020-00854-5 (PMC7257554; doi:10.1186/s40168-020-00854-5)
Supplement: Supplementary file 13 — Additional file 12: The ability of microbiota to convert urea into glutamine was stronger in the gut of FMT_MM mice at week 0, without the induction of MM tumorigenesis. [file 40168_2020_854_MOESM12_ESM.docx]

**Additional file 12:**

In the mouse experiment performed, the results suggested that the nitrogen recycling bacteria are responsible for the progression of MM through conversion of urea into glutamine, which then will be used by the tumor cells to proliferate. However, we analyzed the glutamine levels in the bone marrow at week 6 when the tumor has already progressed. Taking into account the differences in tumor development and how the tumor will affect renal function, more urea will be accumulated in serum, excreted to the intestinal tract and used by the bacteria to produce glutamine and other amino acids. It is possible therefore that the microbiome in MM could be promoting tumor progression through a different mechanism and the changes detected at the end of the experiment are consequence rather than cause of the tumor progression. Thus, to confirm the hypothesis, it will be important to analyze the levels of glutamine production, glutamine synthase and urease activities at week 0, before the tumor has been induced.

Subsequently, we performed supplemental FMT experiments using different stool donors from MM and HC subjects, respectively. The clinical characteristics of fecal donors are presented in **Table 1**. In these experiments, all mice were treated with a cocktail of broad-spectrum antibiotics in the drinking water for 1 week. Then, they were randomly assigned to one of the three groups (PBS, FMT_HC, FMT_MM), and FMT experiments were performed for 2 weeks (twice per week). Subsequently, all mice were euthanized without the induction of MM tumorigenesis. As expected, we detected higher concentrations of glutamine in the serum and cecal contents of FMT_MM mice (**Figure 1a, b**) and higher activities of urease and glutamine synthetase in the cecal contents of FMT_MM mice (**Figure 1c, d**). The results implied that the ability of microbiota to convert urea into glutamine was stronger in the gut of FMT_MM mice.

**Table 1.** The clinical characteristics of fecal donors.

| Fecal donor | Gender | Age (years) | BMI (kg/m2) | Urease (U/L) | Glutamine synthetase (U/L) | Plasma cell ratio (%) | Subtype | ISS stage | DS stage | Urea (mM) | Creatinine (uM) |
| --- | --- | --- | --- | --- | --- | --- | --- | --- | --- | --- | --- |
| MM | male | 58 | 21.05 | 51.23 | 36.49 | 40.5 | IgG κappa | III | IIIA | 4.27 | 92 |
| HC | male | 55 | 24.81 | 8.19 | 6.68 | / | / | / | / | / | / |


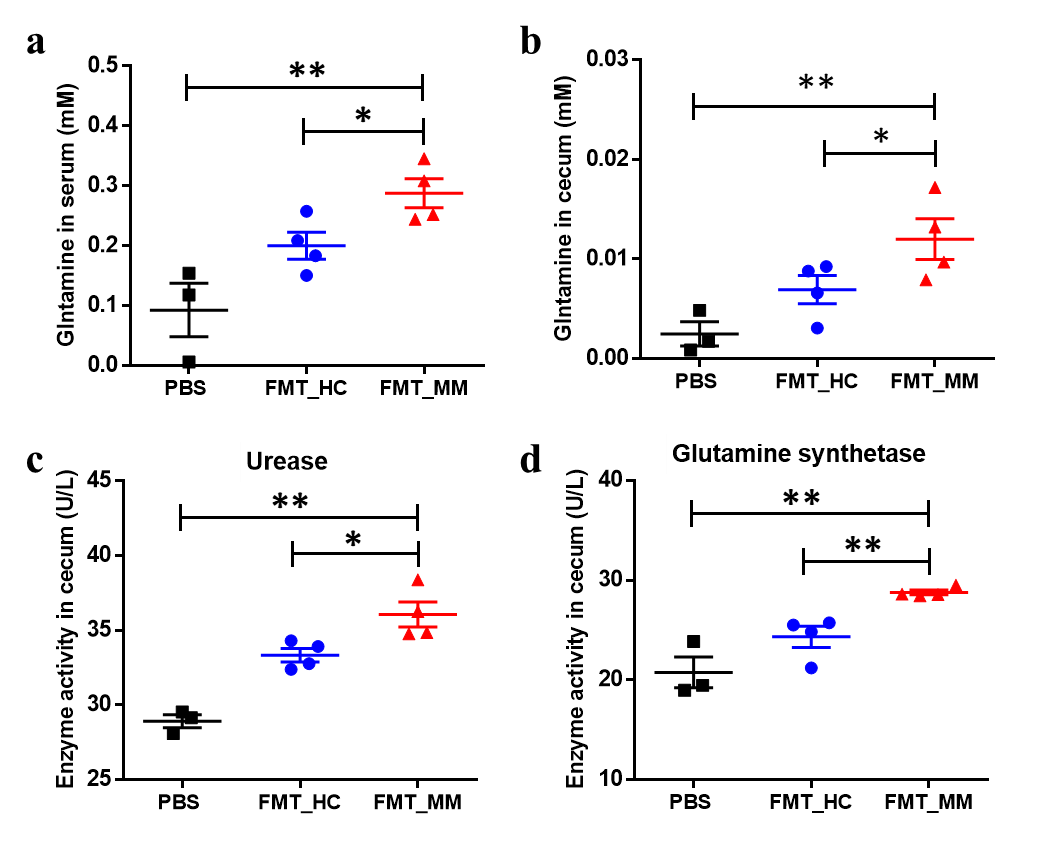


**Figure 1**. FMT mice experiments without inducing MM tumorigenesis. **a,** **b** The concentration of glutamine in the serum (**a**) and cecal contents (**b**) of PBS, FMT_HC, and FMT_MM mice, respectively. **c**, **d** The activities of cecal urease (**c**) and glutamine synthase (**d**) in the cecal contents of PBS, FMT_HC, and FMT_MM mice, respectively. *P*-value was determined by using two-tailed unpaired t-test. * *P*<0.05, ** *P*<0.01, *** *P*<0.001.
