# Supplementary material for: Alterations of gut microbiome accelerate multiple myeloma progression by increasing the relative abundances of nitrogen-recycling bacteria
Source: Microbiome. 2020 May 28;8:74. doi: 10.1186/s40168-020-00854-5 (PMC7257554; doi:10.1186/s40168-020-00854-5)
Supplement: Supplementary file 14 — Additional file 13: The FMT experiment was repeated twice, in which fecal donors from two MM patients and two HCs were separately applied to female or male mice. And the same results were detected as previous experiment, suggesting the experimental results are reproducible. [file 40168_2020_854_MOESM13_ESM.docx]

**Additional file 13:**

In the mouse experiment performed, only one donor was used to analyze the effect of FMT on MM progression in mice. Although the results obtained were the expected ones, it will strength the conclusions of the manuscript to repeat the experiment with other donors in order to show that the result is reproducible. Therefore, we repeated the FMT experiment twice, in which fecal donors from two multiple myeloma (MM) patients and two healthy controls (HCs) were separately applied to mice of different genders (female, male). The clinical characteristics of fecal donors were present in **Table 1**. Here, we detected the same results as in previous experiment (**Figure 1 and Figure 2**), suggesting our experimental results are reproducible.

**Table 1.** The clinical characteristics of fecal donors.

| Fecal donor | Gender | Age (years) | BMI (kg/m2) | Urease (U/L) | Glutamine synthetase (U/L) | Plasma cell ratio (%) | Subtype | ISS stage | DS stage | Urea (mM) | Creatinine (uM) | Mice gender |
| --- | --- | --- | --- | --- | --- | --- | --- | --- | --- | --- | --- | --- |
| MM1 | female | 69 | 15.82 | 25.82 | 24.81 |  | Lamda | III | IIIA | 4.48 | 86 | male |
| HC1 | male | 54 | 22.10 | 12.67 | 12.02 | **/** | **/** | **/** | **/** | **/** | **/** | male |
| MM2 | female | 61 | 19.43 | 13.07 | 15.80 | 26 | Lamda | III | IIIB | 10.67 | 313 | female |
| HC2 | male | 55 | 24.80 | 8.19 | 6.68 | **/** | **/** | **/** | **/** | **/** | **/** | female |


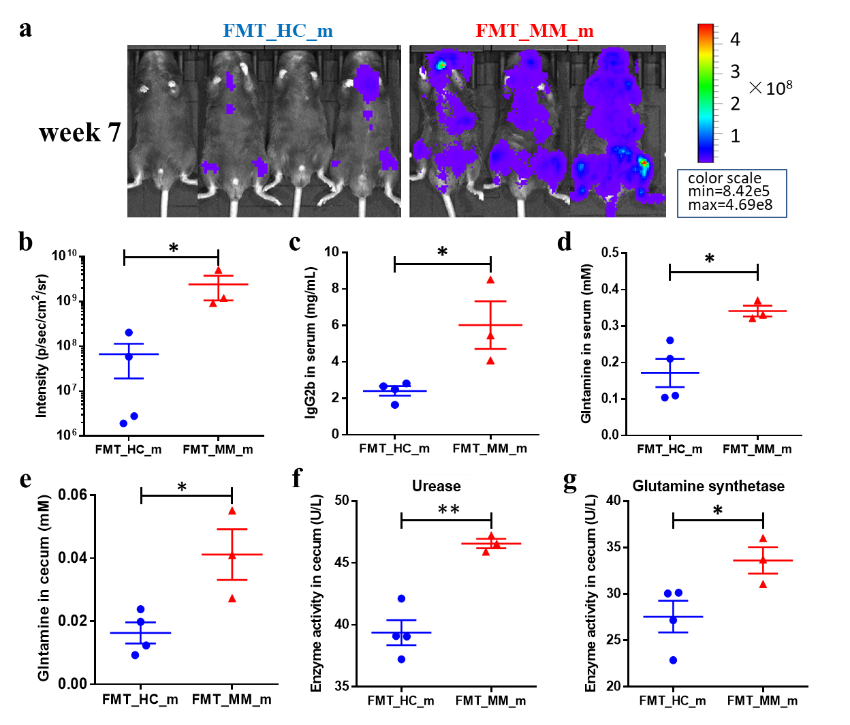


**Figure 1.** Fecal microbiota from MM and HC stool donors, respectively, was transplanted into male mice. **a** Live imaging of experimental mice at week 7. **b, c** The tumor fluorescence intensity (**b**) and serum lgG2b (**c**) of experimental mice at week 7. **d, e** The concentration of glutamine in the serum (**d**) and cecal contents (**e**) of experimental mice at week 7. **f**, **g** The activities of urease (**f**) and glutamine synthetase (**g**) in the cecal contents of experimental mice at week 7. *P*-value was determined by using two-tailed unpaired t-test. * *P*<0.05, ** *P*<0.01, *** *P*<0.001.


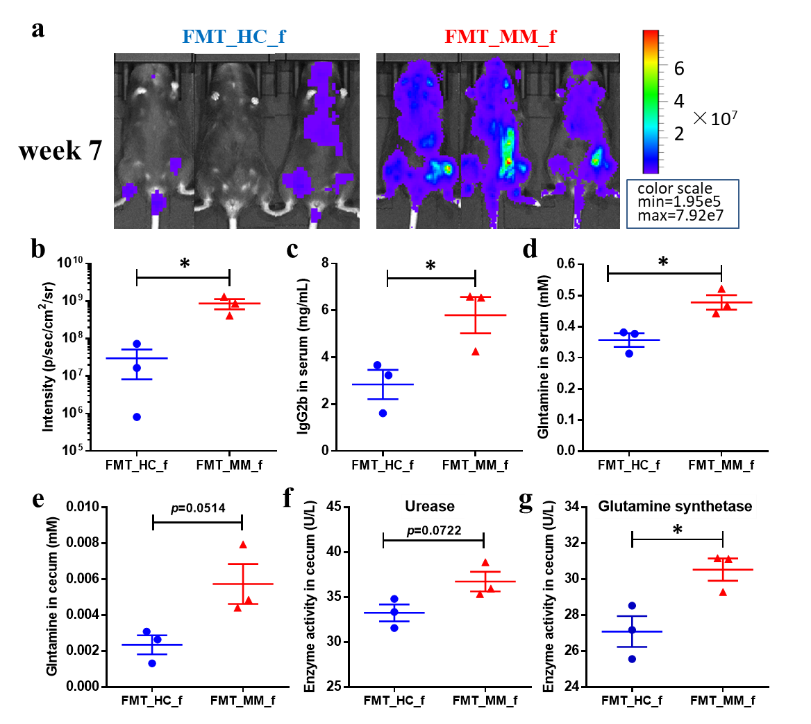


**Figure 2.** Fecal microbiota from MM and HC stool donors, respectively, was transplanted into female mice. **a** Live imaging of experimental mice at week 7. **b, c** The tumor fluorescence intensity (**b**) and serum lgG2b (**c**) of experimental mice at week 7. **d, e** The concentration of glutamine in the serum (**d**) and cecal contents (**e**) of experimental mice at week 7. **f**, **g** The activities of urease (**f**) and glutamine synthetase (**g**) in the cecal contents of experimental mice at week 7. *P*-value was determined by using two-tailed unpaired t-test. * *P*<0.05, ** *P*<0.01, *** *P*<0.001.
