# Supplementary material for: Alterations of gut microbiome accelerate multiple myeloma progression by increasing the relative abundances of nitrogen-recycling bacteria
Source: Microbiome. 2020 May 28;8:74. doi: 10.1186/s40168-020-00854-5 (PMC7257554; doi:10.1186/s40168-020-00854-5)
Supplement: Supplementary file 15 — Additional file 14: Figure S9. The changes in relative abundances of several MM-enriched bacteria. (a) Heatmap reflects the changes of the scaled relative abundance of bacteria over time in NaCl, NH4Cl, and Urea mice. (b) PCoA was performed based on the Euclidean distance calculated from the relative abundances of bacteria at week 0, week 2, week 4, and week 6. [file 40168_2020_854_MOESM14_ESM.docx]

**Additional file 14: Figure S9. The changes in relative abundances of several MM-enriched bacteria.**

(**a**) Heatmap reflects the changes of the scaled relative abundance of bacteria over time in NaCl, NH_4_Cl, and Urea mice.

(b) PCoA was performed based on the Euclidean distance calculated from the relative abundances of bacteria at week 0, week 2, week 4, and week 6.
