# Supplementary material for: Alterations of gut microbiome accelerate multiple myeloma progression by increasing the relative abundances of nitrogen-recycling bacteria
Source: Microbiome. 2020 May 28;8:74. doi: 10.1186/s40168-020-00854-5 (PMC7257554; doi:10.1186/s40168-020-00854-5)
Supplement: Supplementary file 16 — Additional file 15: Table S4. The subjects’ characteristics of metagenome sequencing samples in this study (a). The subjects’ characteristics of un-metagenome sequencing samples in the expanded cohort of this study (b). The characteristics of two MGUS patients in this study (c). [file 40168_2020_854_MOESM15_ESM.docx]

**Supplemental Table 4a. The subjects’ characteristics of metagenome sequencing samples in this study.**

| **Samples' characteristics** | **MM** | **HC** | **P-value** |
| --- | --- | --- | --- |
| Male (%) | 14/19 (73.7) | 13/18 (72.2) | 1**^†^** |
| Age (years) | 60.4±6.2 | 57.9±4.4 | 0.1787**^‡^** |
| BMI (kg/m2) | 22.9±4.2 | 24.3±2.1 | 0.4081**^‡^** |
| Urea (mM) | 11.05 | - | - |
| Creatinine (uM) | 155.3 | - | - |
| **Subtype of MM** |  |  |  |
| lgG | 4/19 (21%) | - | - |
| lgA | 4/19 (21%) | - | - |
| lgD | 1/19 (5%) | - | - |
| Light chain type-kappa | 1/19 (5%) | - | - |
| Light chain type-lamda | 4/19 (21%) | - | - |
| Nonsecretory | 5/19 (26%) | - | - |
| **Plasma cell ratio** |  |  |  |
| <20% | 5/16 (31%) | - | - |
| 20~50% | 4/16 (25%) | - | - |
| >50% | 7/16 (44%) | - | - |
| **ISS stage** |  |  |  |
| I | 0/13 | - | - |
| II | 6/13 (46%) | - | - |
| III | 7/13 (54%) | - | - |
| **DS stage** |  |  |  |
| I | 0/12 | - | - |
| II | 2/12 (17%) | - | - |
| III | 10/12 (83%) | - | - |

Note: **^†^** represents two-tailed Fisher’s exact t-test, **^‡^** represents two-tailed Welch’s t-test.

**Supplemental Table 4b. The subjects’ characteristics of un-metagenome sequencing samples in this study.**

| **Samples' characteristics** | **MM** | **HC** | **P-value** |
| --- | --- | --- | --- |
| Male (%) | 14/17 (82.4) | 16/21 (76.2) | 0.7087**^†^** |
| Age (years) | 57.4±7.0 | 53.5±10.7 | 0.1809**^‡^** |
| BMI (kg/m2) | 23.4±3.1 | 23.7±2.3 | 0.7357**^‡^** |
| Urea (mM) | 5.5 | - | - |
| Creatinine (uM) | 158.8 | - | - |
| **Subtype of MM** |  |  |  |
| lgG | 8/17 (47%) | - | - |
| lgA | 3/17 (18%) | - | - |
| Light chain type-kappa | 2/17 (12%) | - | - |
| Light chain type-lamda | 3/17 (18%) | - | - |
| Nonsecretory | 1/17 (6%) | - | - |
| **Plasma cell ratio** |  |  |  |
| <20% | 5/15 (33%) | - | - |
| 20~50% | 6/15 (40%) | - | - |
| >50% | 4/15 (27%) | - | - |
| **ISS stage** |  |  |  |
| I | 1/15 (7%) | - | - |
| II | 3/15 (20%) | - | - |
| III | 11/15 (73%) | - | - |
| **DS stage** |  |  |  |
| I | 0/14 | - | - |
| II | 3/14 (21%) | - | - |
| III | 11/14 (79%) | - | - |

Note: **^†^** represents two-tailed Fisher’s exact t-test, **^‡^** represents two-tailed Welch’s t-test.

**Supplemental Table 4c. The characteristics of two MGUS patients in this study.**

|  | **Gender** | **Age (year)** | **BMI (kg/m2)** | **Plasma cell ratio** | **Subtype of MM** |
| --- | --- | --- | --- | --- | --- |
| MGUS_01 | Female | 61 |  | 7.2 | lgM lamda |
| MGUS_02 | Female | 42 |  | 5.5 | **/** |
