# Supplementary material for: Alterations of gut microbiome accelerate multiple myeloma progression by increasing the relative abundances of nitrogen-recycling bacteria
Source: Microbiome. 2020 May 28;8:74. doi: 10.1186/s40168-020-00854-5 (PMC7257554; doi:10.1186/s40168-020-00854-5)
Supplement: Supplementary file 17 — Additional file 16: Table S5. The list presents the primers used in this study. [file 40168_2020_854_MOESM16_ESM.docx]

**Supplemental Table 5. The list presents all primers in this study.**

| Primer | NR number | Sequence (5′-3′) | Product size (bp) | Coverage (%) |
| --- | --- | --- | --- | --- |
| *Anaerostipes hadrus* | NR_117139.2 | F-TCTTCGGAACTGAAGATTTGGTGA | 152 | 0.1 |
|  |  | R-ACCACCGGAGTTTTTACCCC |  |  |
| *Bifidobacterium catenulatum* | NR_041875.1 | F-TGGCGAACGGGTGAGTAATG | 158 | 0 |
|  |  | R-CCGCCGACTACCTGATAGGA |  |  |
| *Bifidobacterium kashiwanohense* | NR_112779.1 | F-CTTGCTCCTGGGTGAGAGTG | 171 | 0 |
|  |  | R-CATCAAGCTGATAGGACGCGA |  |  |
| *Bifidobacterium pseudocatenulatum* | NR_037117.1 | F-GGGATCCATCAGGCTTTGCT | 166 | 0 |
|  |  | R-ACCCCATCCCATACCGATGA |  |  |
| *Butyrivibrio hungatei* | NR_025525.1 | F-AAATCTTAGTGGCGGACGGG | 171 | 0 |
|  |  | R-ACCCTGCCAACTGGCTAATC |  |  |
| *Clostridioides difficile* | NR_112172.1 | F-TACTTCGGTAAAGAGCGGCG | 162 | 0.1 |
|  |  | R-TAATCAGACGCGGGTCCATC |  |  |
| *Clostridium butyricum* | NR_112170.1 | F-GCGATGAAGCTCTTCGGGA | 221 | 0 |
|  |  | R-CACCAACTAGCTAATGCGACG |  |  |
| *Clostridium beijerinckii* | NR_113388.1 | F-CAAGTCGAGCGATGAAGCTC | 180 | 0 |
|  |  | R-GACGCGGGTCCATCTCATAG |  |  |
| *Clostridium saccharobutylicum* | NR_122061.1 | F-TCTTCGGAAGTGGATTAGCGG | 174 | 0 |
|  |  | R-AACTAGCTAATGCGACGCGG |  |  |
| *Clostridium saccharoperbutylacetonicum* | NR_102516.1 | F-CAAGTCGAGCGATGAAGTTCC | 180 | 0 |
|  |  | R-GACGCGGGTCCATCTCATAG |  |  |
| *Fusobacterium varium* | NR_113384.1 | F-GATCCTTCGGGTGAAGGTGG | 189 | 0 |
|  |  | R-TGAGCCGTTACCTCACCAAC |  |  |
| *Herbinix luporum* | NR_152095.1 | F-AGCAGGGAAGAAAATGACGGT | 203 | 0 |
|  |  | R-ACCTCTCCTGCACTCTAGCA |  |  |
| *Lachnoclostridium phytofermentans* | NR_074652.1 | F-GGAAGTCCTCGGATGGAAGTT | 174 | 0 |
|  |  | R-AGACGCGGGTCCATCTCATA |  |  |
| *Paeniclostridium sordellii* | NR_112173.1 | F-AGTAACGCGTGGGTAACCTG | 172 | 0 |
|  |  | R-GATCGTTGCCTTGGTAAGCC |  |  |
| *Streptococcus pasteurianus* | NR_043660.1 | F-GAGTTGCGAACGGGTGAGTA | 175 | 0.4 |
|  |  | R-CCGTTACCCCACCAACTAGC |  |  |
| *Bifidobacterium dentium* | NR_037115.2 | F-GAGAGTGGCGAACGGGTG | 168 | 0 |
|  |  | R-TTACCCCGCCATCAAGCTG |  |  |
| *Citrobacter freundii* | NR_028894.1 | F-GAGCTTGCTCCTTGGGTGAC | 189 | 0.2 |
|  |  | R-AGCCGTTACCCCACCTACTA |  |  |
| *Collinsella aerofaciens* | NR_028604.1 | F-CTCTCCGGAGGGAAGCGA | 175 | 0 |
|  |  | R-GCCGTCTACCTGATGGGC |  |  |
| *Enterobacter cloacae* | NR_102794.2 | F-ATGCAAGTCGAACGGTAGCA | 151 | 0.7 |
|  |  | R-GGTCCCCCTCTTTGGTCTTG |  |  |
| *Intestinimonas butyriciproducens* | NR_118554.1 | F-CGGACAACGAAAGGGAATGC | 168 | 0 |
|  |  | R-CAGACGCGAGTCCATCTCAG |  |  |
| *Klebsiella aerogenes* | NR_118556.1 | F-TGCCTGATGGAGGGGGATAA | 182 | 3.7 |
|  |  | R-GTGGCTGGTCATCCTCTCAG |  |  |
| *Klebsiella pneumoniae* | NR_119278.1 | F-GCGGACGGGTGAGTAATGTC | 164 | 2.2 |
|  |  | R-AGCCGTTACCCCACCTACTA |  |  |
| *Klebsiella variicola* | NR_025635.1 | F-TGCCTGATGGAGGGGGATAA | 166 | 3.7 |
|  |  | R-TCAGACCAGCTAGGGATCGT |  |  |
| *Prevotella ruminicola* | NR_044632.1 | F-AACACATGCAAGTCGAGGGG | 199 | 0.1 |
|  |  | R-TCAGACGCATCCCCATCCTA |  |  |
| *Prevotella melaninogenica* | NR_102895.1 | F-CACATGCAAGTCGAGGGGAA | 186 | 0 |
|  |  | R-GCATCCCCATCCATTACCGA |  |  |
| *Raoultella ornithinolytica* | NR_114502.1 | F-AGCGGTAGCACAGAGAGC | 163 | 0.5 |
|  |  | R-ATCTGATGGCATGAGGCCC |  |  |
| *Streptococcus anginosus* | NR_118289.1 | F-AGACTGTGAGTTGCGAACGG | 156 | 0 |
|  |  | R-AACGCAGGTCCATCTACTAGC |  |  |
| *Streptococcus mitis* | NR_115732.1 | F-GAGTTGCGAACGGGTGAGTA | 147 | 0.8 |
|  |  | R-CGCAGGTCCATCTGGTAGTG |  |  |
| *Streptococcus parasanguinis* | NR_024842.1 | F-CGCTGAAGCTTGGTGCTTG | 182 | 0 |
|  |  | R-ACAACGCAGGTCCATCTCTT |  |  |
| *Streptococcus pneumoniae* | NR_028665.1 | F-GAACGCTGAAGGAGGAGCTT | 179 | 0.5 |
|  |  | R-CGCAGGTCCATCTGGTAGTG |  |  |
| *Streptococcus salivarius* | NR_042776.1 | F-ACGCTGAAGAGAGGAGCTTG | 180 | 0.1 |
|  |  | R-ACGCAGGTCCATCTTGTAGTG |  |  |
| *Streptococcus thermophilus* | NR_042778.1 | F-ACGCTGAAGAGAGGAGCTTG | 180 | 0.1 |
|  |  | R-ACGCAGGTCCATCTTGTAGTG |  |  |
| *Streptococcus oralis* | NR_114413.1 | F-CGCTGAAGCTTGGTGCTTG | 178 | 0.1 |
|  |  | R-CGCAGGTCCATCTGGTAGTG |  |  |
| *Streptococcus gordonii* | NR_115242.1 | F-ACCATAGTACGCTTTGGAAACTG | 218 | 0 |
|  |  | R-GGGCCTAACACCTAGCACTC |  |  |
| Total Bacteria* |  | F-GTGSTGCAYYGGYTGTCGTCA |  | 73.8 |
|  |  | R-ACCGTCRTCCMCACCTTCCTC |  |  |

***:** ‘Total Bacteria’ represents the primer which was used to amply total bacteria ^[1]^.

**Reference**

[1] Maeda H, et al. Quantitative real-time PCR using TaqMan and SYBR Green for Actinobacillus actinomycetemcomitans, Porphyromonas gingivalis, Prevotella intermedia, tetQ gene and total bacteria. FEMS Immunology & Medical Microbiology 39, 81-86 (2003).
