# Supplementary material for: Alterations of gut microbiome accelerate multiple myeloma progression by increasing the relative abundances of nitrogen-recycling bacteria
Source: Microbiome. 2020 May 28;8:74. doi: 10.1186/s40168-020-00854-5 (PMC7257554; doi:10.1186/s40168-020-00854-5)
Supplement: Supplementary file 19 — Additional file 18: Figure S10. Construction and verification of glnA-mutant Klebsiella pneumoniae by homologous recombination technique using plasmid pKO3-Km. (a) Schematic depiction of glnA sequencing, in which fragment in blue was deleted. (b) Schematic diagram of plasmid pKO3-Km. (c) Primers used for glnA disruption and glnA qPCR were designed according to the Klebsiella pneumoniae subsp. HS11286 chromosome sequence (NC_016845.1: c36644-35196) and plasmid pKO3-Km sequence. (d) Agarose gel for PCR products, the left panel shows that with Klebsiella pneumoniae genomic DNA as the template; the right panel shows that with the mix left PCR products as a template. (e) Agarose gel for PCR products of 10 clones of pKO3-km-glnAmut. (f) NCBI Blast sequence alignment for pKO3-km-glnAmut. (g) The relative abundance of gene glnA in the clones Mut1 to 10 and wild-type Klebsiella pneumoniae using qPCR. (h) The remaining concentrations of glutamine in the broth. Blk represents the initial concentration. [file 40168_2020_854_MOESM18_ESM.docx]

**Additional file 18: Figure S10**. Construction and verification of *glnA*-mutant *Klebsiella pneumoniae* by homologous recombination technique using plasmid pKO3-Km. **a** Schematic depiction of *glnA* sequencing, in which fragment in blue was deleted. **b** Schematic diagram of plasmid pKO3-Km. **c** Primers used for *glnA* disruption and *glnA* qPCR were designed according to the *Klebsiella pneumoniae* subsp. HS11286 chromosome sequence (NC_016845.1: c36644-35196) and plasmid pKO3-Km sequence. **d** Agarose gel for PCR products, the left panel shows that with *Klebsiella pneumoniae* genomic DNA as the template; the right panel shows that with the mix left PCR products as a template. **e** Agarose gel for PCR products of 10 clones of pKO3-km-glnA^mut^. **f** NCBI Blast sequence alignment for pKO3-km-glnA^mut^. **g** The relative abundance of gene *glnA* in the clones Mut1 to 10 and wild-type *Klebsiella pneumoniae* using qPCR. **h** The remaining concentrations of glutamine in the broth. Blk represents the initial concentration.
